# Efficacy of Diet on Quality of life in Multiple Sclerosis (EDQ-MS): a study protocol for a randomized controlled clinical trial

**DOI:** 10.1186/s13063-025-09157-2

**Published:** 2025-10-27

**Authors:** Farnoosh Shemirani, Anna M. Klein, Allison R. Groux, Rachael Kilpatrick, Lisa Brooks, Mary A. Ehlinger, Warren G. Darling, Vincent A. Magnotta, Christine M. Gill, Karin F. Hoth, Ashutosh Mangalam, Patrick Ten Eyck, Arturo S. Martinez, Jordan Hook, Tyler J. Titcomb, Linda G. Snetselaar, Terry L. Wahls

**Affiliations:** 1 Department of Internal Medicine, University of Iowa, Iowa City, IA USA; 2 Department of Health and Human Physiology, University of Iowa, Iowa City, IA USA; 3 Department of Radiology, University of Iowa, Iowa City, IA USA; 4 Department of Neurology, University of Iowa, Iowa City, IA USA; 5 Department of Psychiatry, University of Iowa, Iowa City, IA USA; 6 Department of Pathology, University of Iowa, Iowa City, IA USA; 7 Institute for Clinical and Translational Science, University of Iowa, Iowa City, IA USA; 8 Department of Epidemiology, University of Iowa, Iowa City, IA USA; 9 Department of Dietetics and Nutrition, Medical Center, University of Kansas, Kansas City, KS USA; 10 Department of Neurology, Medical Center, University of Kansas, Kansas City, KS USA

**Keywords:** Multiple sclerosis, Modified paleolithic elimination diet, Ketogenic diet, Time-restricted feeding, Quality of life

## Abstract

**Background:**

The primary objective of this study is to evaluate the efficacy of two dietary interventions, time-restricted olive oil-based (TROO) ketogenic and modified Paleolithic elimination (MPE), compared to usual care control group receiving Dietary Guidelines for Americans (DGA) resources, on quality of life (QoL) among individuals with relapsing–remitting multiple sclerosis (RRMS). The secondary objectives are to evaluate the long-term effects on functional disability, fatigue, mood, motor, ocular and cognitive function, and disease activity.

**Methods:**

The proposed study will consist of 162 study participants attending 3 in-person site visits at months 0, 3, and 24, with online surveys every 3 months. This study will use a randomized, single-blind, controlled design to investigate the short-term (6 months) and long-term (24 months) efficacy of intervention diets on QoL and related outcomes stated above. On-site study visits will include motor, ocular and cognitive function, as well as magnetic resonance analyses. Blood and saliva biospecimens will be collected and frozen for future metabolome and microbiome analyses, respectively. Participants assigned to TROO keto and MPE diets will be given diet-specific educational materials and dietary supplements. Usual care control group will be given DGA resources and monthly educational resources. Dietary assessments will be collected by the Diet History Questionnaire III (DHQ III) at months 0, 3, 12 and 24. Registered Dietitian Nutritionists (RDNs) will deliver the dietary intervention to the TROO and MPE groups, using Social Cognitive Theory (SCT), Self-Determination Theory, and Motivational Interviewing (MI) techniques to facilitate effective behavior change.

**Discussion:**

There is an unmet need for rigorous randomized controlled trials to assess the efficacy of dietary interventions on MS-related symptoms and disease activity. The findings from this study will provide necessary information regarding the role of specific dietary interventions in MS care.

**Trial registration:**

ClinicalTrials.gov, NCT05007483. Registered on August 16, 2021. https://clinicaltrials.gov/study/NCT05007483.

**Supplementary Information:**

The online version contains supplementary material available at 10.1186/s13063-025-09157-2.

## Introduction

### Background and rationale {6a}

Multiple sclerosis (MS) is a chronic, devastating neurodegenerative disease characterized by demyelination of neurons [1]. Disease modifying therapies (DMTs) are the current standard of care, and they are efficacious for reducing relapses, disability progression, and disease activity [2, 3]. However, since pharmacological treatment options are limited for other symptoms of MS including impaired cognitive function [4], many people with MS seek additional therapies such as diet modifications [5]. In fact, up to 50% of people with MS report implementing dietary modifications to reduce their symptoms and improve the overall quality of life (QoL) [6, 7]. A recent network meta-analysis showed that many specific dietary interventions reduce fatigue and improve QoL among people with MS [8]; however, the current state of evidence does not support recommending a specific dietary pattern in MS [9], mainly pertaining to a lack of evidence for the effect of diet on disease activity and unknown mechanisms by which diet impacts disease outcomes [10–12].

Results from several studies suggest that diet quality is inversely associated with symptoms of MS, including disability [13], fatigue, depression, and anxiety [14], and QoL [15]. Furthermore, a recent pilot case–control study among 22 people with MS over a period of 10 years showed those who had followed the diet and lifestyle change program for longer than 10 years had white matter lesion (WML) volumes three times smaller compared to people with MS who had not followed the program [16]. Additionally, evidence suggests that a pro-inflammatory diet, indicated by a higher Dietary Inflammatory Index (DII) score, is associated with a higher risk of relapse and increased fluid-attenuated inversion recovery (FLAIR) lesion volume in people with MS [17].

Two dietary approaches with supporting preliminary evidence are the ketogenic diet [18–20] and Modified Paleolithic Elimination (MPE) diet [21–23]. A previous randomized, parallel-arm trial, comparing these two diets among people with relapsing–remitting MS (RRMS), showed that the MPE diet group experienced statistically and clinically significant within-group improvements in mental and physical QoL [24]. Furthermore, a separate single-arm study demonstrated that a ketogenic diet improved fatigue and depression. Moreover, those following the ketogenic diet did not show any change in disease activity at 6 months as demonstrated by brain magnetic resonance imaging (MRI) [18].

Findings from a previous pilot randomized controlled trial comparing the effects of a modified medium chain triglyceride (MCT)-based ketogenic and an MPE diet on fatigue and QoL showed significant improvement in clinical outcomes following the MPE diet, while no clinical outcome significantly changed following MCT-based ketogenic diet [22]. Of note, despite good adherence, not all participants consuming the MCT-based ketogenic diet achieved nutritional ketosis. The current study has initiated the exploration of the advantages of combining time-restricted eating (TRE) with an olive oil-based ketogenic diet to improve the maintenance and longevity of nutritional ketosis. Importantly, recent pilot studies have shown that TRE is feasible and acceptable among people with RRMS [25, 26]. Given similar mechanisms of action for both diet strategies, there has been some evidence of benefits of applying combined strategy to manage chronic disorders [27]. However, more large-scale studies are needed to further our understanding of the combined application of ketogenic and fasting diets in the context of MS [27].

Overall, previous studies exploring the effects of dietary changes on MS outcomes were constrained by the absence of a randomized control group, short follow-up durations, and a lack of MRI monitoring for disease activity. Thus, these studies could not establish efficacy of dietary interventions for MS-specific outcomes. Therefore, this study aims to assess the efficacy of the MPE, and time-restricted olive oil-based (TROO) ketogenic diets compared to a usual care control provided with educational information from the 2020 Dietary Guidelines for Americans (DGA) among a diverse cohort of people diagnosed with RRMS over a 24-month period. This trial will make a significant contribution to understanding the effects of diet on MS-specific outcomes by bridging gaps in the scientific literature regarding the effect of diets on MS disease activity and providing health care providers with evidence-based recommendations regarding the use of diet as an adjunct therapy in MS patient care.

### Objectives {7}

The primary objective of this study is to evaluate the efficacy of two dietary interventions, the TROO ketogenic and MPE diets on MS QoL compared to a usual care control group provided with information from the DGA. The secondary objectives of the study are to evaluate the long-term effects on functional disability, fatigue, mood, motor, ocular and cognitive function, and disease activity, as assessed by brain MRI.

### Trial design {8}

The EDQ-MS study will use a randomized, single-blind, controlled design to investigate the short-term (6 months) and long-term (24 months) efficacy of the intervention diets on QoL and related outcomes compared to a usual care control group.

## Methods: participants, interventions and outcomes

### Study setting {9}

Data collection will be completed at the University of Iowa (UI) Clinical Research Unit (CRU) in Iowa City, IA, USA, by trained research assistants blinded to the group assignments. Participants will be instructed to not discuss their diet with the research assistants.

### Eligibility criteria {10}

The inclusion criteria include:

1. A definitive diagnosis of RRMS based on the 2017 revised McDonald Criteria [28].
2. The ability to prepare, or availability of another person to prepare, home-cooked meals.
3. Must own a computer, smartphone, or tablet device with internet access to complete online surveys and capable of running study-related applications.
4. Must reliably attend scheduled visits with the study team (including virtual and site visits) and follow study procedures.
5. Be between the ages of 18 and 70 at the time of consent.
6. Must be able to walk 25 feet without support OR with only unilateral support from a cane or walking stick (ankle/foot orthotic is allowed).
7. Willingness to be randomized and follow any of the study diets.
8. Must consent to sharing the clinical notes from their primary care and neurology providers during the study period.
9. Must live or reside in North American countries (United States, Canada, or Mexico).

The exclusion criteria include:

1. Moderate or severe cognitive impairment, as assessed by Short Portable Mental Status Questionnaire (SPMSQ), and defined as more than four errors.
2. Use of insulin, warfarin, or weight loss medications such as orlistat that cause fat malabsorption.
3. Worsening of symptoms resulting in the initiation or change of treatment including steroids (e.g., solumedrol, prednisone) or DMTs within 4 weeks prior to consent.
4. Treatment for cancer by radiation or chemotherapy within 12 months prior to consent, other than skin cancer.
5. Diagnosis of clinically significant heart disease, liver disease, kidney disease, or history of oxalate kidney stones.
6. Diagnosis of type II diabetes that does not have approval from treating physicians to adopt any of the three study diets.
7. Clinical diagnosis of a psychiatric condition that may impact study adherence (e.g., schizophrenia, bi-polar disorder, severe depression and/or anxiety).
8. An active eating disorder such as anorexia, bulimia, binge eating, or orthorexia.
9. Measurement of Body Mass Index (BMI) < 20.
10. Confirmation of pregnancy or planning to become pregnant within the next 2 years.
11. History of diagnosed fat intolerance/malabsorption such as cholecystectomy or uncontrolled exocrine pancreatic insufficiency.
12. Participation in another research study investigating MS treatments, diet, or exercise.
13. Presence of a contraindication to completing a brain MRI or having claustrophobia which interferes with completion of MRI studies without the use of sedation.

### Who will take informed consent? {26a}

Prior to enrollment, prospective participants will first complete a pre-enrollment Zoom call with a member of the study team. This call will be used to verify study eligibility criteria and administer the Short Portable Mental Status Questionnaire (SPMSQ) to assess cognitive ability. Eligible participants will be emailed a link to review the Institutional Review Board (IRB)-approved consent form and consent summary, which they may discuss with family or friends if desired. A separate consent review call will then be conducted via Zoom, during which a study team member will explain the consent in full, including the study’s purpose, procedures, participation requirements, potential risks, and participants’ rights. Participants will be informed that participation is voluntary and that they may withdraw from the study at any time without prejudice. Additionally, a medical history review call will be conducted via Zoom prior to the site visit to ensure there are no medical conditions that would exclude participation. At the baseline site visit, any remaining questions will be addressed before participants are asked to sign and date the consent form. A paper or scanned PDF copy of the signed consent will be provided for their records.

### Additional consent provisions for collection and use of participant data and biological specimens {26b}

The informed consent includes permission for the use of collected data, medical records, as well as biological specimens for future use (Additional file 1).

## Interventions

### Explanation for the choice of comparators {6b}

Since nutrition counseling is not a current clinical recommendation for individuals with MS, the usual care control group will not receive diet education from the study RDN. Instead, they will receive educational materials and resources from the 2020–2025 DGA and be allowed to utilize the resources to any degree of compliance. The rationale for providing only educational materials on the DGA to the usual care control group is that: 1) nutrition counseling and education provided by RDNs is not part of the standard of care for MS in the U.S. and 2) that people with MS are recommended to follow a general healthy diet such as the DGA. Giving study participants educational material on the DGAs and the choice to follow the DGA recommendations to any degree of compliance maintains clinical equipoise of the usual care control.

### Intervention description {11a}

The intervention arms in this study include the MPE diet (Intervention Group 1) and TROO ketogenic diet groups (Intervention Group 2). Participants will be implementing their randomly assigned intervention diet for 24 months. Participants in the two intervention arms will receive education and resources from the study RDNs for their assigned diet and receive continuous support from the study team to facilitate their implementation and adherence to their study diet. Participants assigned to either intervention group will have the opportunity to attend monthly group nutrition counseling sessions led by RDNs. Additional continuous support will include scheduled coaching calls via Zoom after starting the assigned diet to address potential questions. Furthermore, participants in the intervention arms will have access to the UI’s Canvas Classroom, which will serve as a focus group and discussion platform.

### Study diets

Key recommendations for each intervention diet are detailed in Table 1. Additionally, specific considerations for each intervention group are outlined as follows.

**Table 1:**
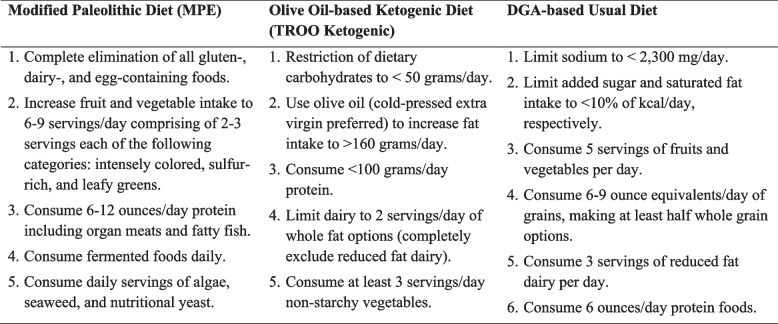
Key diet components/recommendations for the MPE, TROO ketogenic, and DGA-based usual diets

#### Elimination and re-introduction phase (MPE group only)

At baseline (month 0), study participants assigned the MPE diet will be instructed to eliminate gluten, dairy, and eggs from their diet. In addition, at visit 2 (month 3), participants will also be instructed to eliminate all grains (including gluten-free), all legumes including peanuts and soy, and nightshade vegetables including tomatoes, potatoes, eggplants, and peppers for 3 months. After 3 months, study participants will be allowed to re-introduce one ingredient at a time, one ingredient per week. Study participants will be asked to keep a symptom diary and record any worsening mental or physical symptoms. If there are foods identified that cause a worsening of symptoms the study participant may remove the food item again. Participants may choose to re-introduce the food again if they desire to confirm whether the food is associated with worsening mental or physical symptoms. If symptoms again recur, the food may be eliminated from their diet for the remaining duration of the study.

#### Ketone monitoring (TROO ketogenic group only)

Participants randomized to the TROO ketogenic diet will be provided a Keto-Mojo® blood ketone and glucose monitor for diet tracking purposes. Participants will be instructed to check blood ketones daily for 2 weeks or until they are firmly in ketosis. After 2 weeks, study participants will be allowed to reduce the frequency of checking ketones to 2 times per week. All testing supplies will be provided, including lancets and test strips, to provide immediate results on blood ketone values. The participants who are assigned to the TROO ketogenic diet will download the Keto-Mojo® application onto their smart phone and create a MyMojoHealth Account for themselves with the assistance of the study team. The study team will teach the participant how to test for ketones using the ketone meter and will show the participant how to synchronize the ketone readings from their ketone meter with their MyMojoHealth account. The participant will be asked to join the study team practice accounts so their assigned RDNs may access the data throughout the duration of the study.

#### Usual care control group

At the initial visit, RDNs will assist the participant in downloading USDA Start Simple with MyPlate application onto their phone or device. RDNs will also review the DGA guidelines and resources available on the DGA website.

### Dietary supplements

Participants will be asked to continue taking any dietary supplements they report at beginning of the study. Participants with suboptimal plasma vitamin D levels will be referred to their treating physicians. The study team will provide fish oil, essential fatty acids, and phosphatidylcholine to participants assigned to the intervention groups only. Participants assigned the intervention diets that are taking fish oil, phosphatidylcholine, or omegas 3 and 6 at baseline will be asked to replace their current supplements with the brand (Pure Encapsulations) being used as part of the study. Bottles will be dispensed at months 0 (baseline visit), 3 (visit 2), and month 12.

The study team will ask study participants to return or report the number of empty supplement bottles that were dispensed at month 24 (end of study visit). If a study participant has a flaxseed or safflower allergy, they will not be asked to take the study essential fatty acid supplement. If the study participant has a known soy allergy, they will not be asked to take the phospholipid complex.

### Dietary education and resources

#### Intervention diet groups

Study participants will receive a food guide developed by study staff that outlines key features of their assigned study diet and given access to educational videos following randomization at the end of their baseline study visit. Participants will be asked to schedule a Zoom video conference session with the RDNs to review the diet intervention. The study participant will be encouraged to review the food guide and diet education videos prior to the Zoom meeting so they are familiar with the information and can generate questions to be asked during the session. Participants will also be given access to a series of educational videos about food shopping and cooking for additional information.

### Usual care control group

Participants assigned to the usual care control group will maintain their current dietary habits and receive educational materials based on the 2020–2025 DGA. To enhance accessibility to these resources, participants will be guided to install the USDA Start Simple with MyPlate mobile application on their phones. Additionally, study participants randomized to the usual care group will receive monthly emails and/or text messages to receive resources with articles, websites, recipes, DGA education materials, and recent MS-related research that does not involve diet.

### Study applications

#### Intervention groups

The study participants are provided with additional resources, including the Canvas Classroom, the MyCap application (for monitoring diet adherence), and the EDQ-MS application (which includes recipes for intervention groups). Participants will be asked to answer three questions about diet-specific components and perceived adherence in the MyCap app. The purpose of the EDQ-MS app is to provide assistance, resources, and support for meal planning and food shopping during the study. Further details about these resources are available in Additional file 2.

#### Usual care group

The study RDNs will assist participants in downloading the USDA Start Simple with MyPlate application onto their phone or device and initial use of the application. This app provides practical tools and resources aligned with the DGA, offering recipes, personalized nutrition guidance, and tips for maintaining a healthy diet (Additional file 2).

#### Nutrition counselling

Participants will have the opportunity to review the diet food guide for either the MPE diet or TROO ketogenic diet with the study dietitian via Zoom video conference. Dietary training to assist the participant in implementing the study diet will be given by an RDN who has experience with the study diet. The food guides provide prompts to eat the desired food groups and to avoid foods that are excluded in both intervention diets. Additional coaching calls will be arranged between the participants and the study team via telephone or Zoom. Two initial coaching calls following the nutrition counselling will be conducted 3–4 days after the Zoom meeting, as well as 7 days after the first call to answer questions as participants begin following their diet.

The conceptual basis for this intervention is informed by Social Cognitive Theory (SCT), which recognizes both the personal and environmental factors that influence health behavior change [29]. Personal factors addressed in SCT include: 1) behavioral capability, which is the understanding and skill to perform a behavior; 2) outcome expectancy, which is assigning a value to the outcomes of behavior change; 3) self-control, which is regulating and monitoring individual behavior; 4) self-efficacy, which refers to a person’s confidence in their ability to successfully perform a behavior; and 5) self-regulation, which facilitates people’s ability to control their behavior. Through this, individuals may create, alter, and commit to goals to achieve a desired outcome. The constructs of SCT will drive the sessions offered to our MS participants. Nutrition counseling techniques based on SCT, Self-Determination Theory [30], and Motivational Interviewing (MI) [31] will also be used to facilitate effective behavior change [32]. SDT focuses on intrinsic motivation and the factors that contribute to it, such as autonomy, competence, and relatedness, and suggests that providing opportunities for individuals to make choices (autonomy), develop skills and capabilities (competence), and foster positive relationships (relatedness) can lead to increased self-efficacy [33].

### Monthly support

#### Usual care control group

During follow-up, participants in the usual care control group will receive monthly text messages, sent by Study Coordinator on behalf of the PI, along with emails containing recipes, cooking tips, and general information from DGA resources, as well as general information about MS. Brief video messages from the PI are provided as needed for participant re-engagement. In addition, engagement texts and emails tied to holidays, participant birthdays, and key study milestones will be sent. These monthly contacts and engagement messages are intended to support participant retention.

### Intervention groups

#### Group support sessions

Both the TROO ketogenic and MPE diet groups will receive monthly group nutrition counseling sessions from the intervention RDNs to facilitate long-term diet adherence. Each intervention group will receive monthly live group behavioral support calls, using motivational interviewing, celebrating participant successes, addressing participants’ diet-related questions, to maintain participant engagement in the study. Participants will be invited to submit questions and comments ahead of time so that their concerns can be discussed even if they cannot attend live calls. The study PI will review questions submitted by the study participants in each group and create videos (specific to the intervention group) that address submitted questions. The calls will be recorded and hosted in the Canvas discussion platform. Monthly calls are expected to increase engagement but making them optional and hosting the recorded live coaching calls in the Canvas discussion platform reduces participant burden. Engagement texts and emails tied to holidays and participant birthdays and key study milestones will also be sent. Monthly contacts and engagement contacts are anticipated to enhance participant retention.

### Criteria for discontinuing or modifying allocated interventions {11b}

No modification of the interventions is anticipated throughout the trial. Reasons for discontinuation of study intervention include:

1. *Cognitive impairment*: If the study participant demonstrates difficulty following or remembering study tasks, a member of the study team may repeat the SPMSQ. If a participant is identified as potentially having cognitive impairment, as assessed by the SPMSQ, and four or more errors are recorded, the study staff will notify the PI to discuss the participant’s cognitive state. The study staff will also send a letter to the participant’s neurologist to inform them that a potential decline in cognition has been observed with a recommendation to evaluate the participant’s cognition and competence as they deem appropriate. The neurologist will be informed if more than four errors were documented, which is a medical stop point. If the neurologist determines that the participant is cognitively impaired, the participant will not be allowed to continue in the study; however, if the treating neurologist reports that the patient is cognitively competent, the participant may continue the study intervention. If the participant’s SPMSQ score falls in the moderate range (5–7 errors) or if the participant’s neurologist deems them to have significant cognitive impairment, the participant will be instructed to discontinue the study intervention, although data collection will continue. If in the severe range (8–10 errors), the participant will stop all study activities and withdraw from the study because the data is considered unreliable.
2. *Physical impairment*: If a study participant becomes physically impaired due to injury, an adverse event, loss of function due to MS or other diagnoses, they may still participate in the study intervention. Other study procedures such as motor and ocular assessments will be performed dependent on the task and impairment as appropriate. The changes in function will be captured in the extenuating circumstances electronic Case Report Form (eCRF) and adverse event forms in the database.
3. *Pregnancy:* If a study participant becomes pregnant during the study, it will be documented in the REDCap data capture platform on the extenuating circumstances eCRF. The study participant will be instructed to follow the diet that is recommended by the medical team that is providing the participant’s pregnancy-related care. The participant may continue to complete other study procedures.
4. *Treating physician no longer recommends*: If a study participant’s treating physician determines it is no longer in the participant’s best interest to continue with some or all of the study interventions, the participant may remain in the study and complete other study procedures. A participant’s treating physician may direct their patient to discontinue or withdraw from the study intervention if any clinical adverse event (AE), laboratory abnormality, or other medical condition or situation occurs such that continued participation in the study would not be in the best interest of the participant.
5. *Participant discontinues on their own*: If a study participant discontinues part or all of the study intervention on their own, they may remain in the study and complete other study procedures. The study team will record when the study intervention was altered or discontinued via patient report.

### Strategies to improve adherence to interventions {11c}

Participants in the MPE diet group will be considered adherent to their diet if DHQ-III survey indicate that they consumed within 20% of recommendations for key intervention diet components (≥ 5 cup equivalent servings of total fruit and vegetables). Adherence to the TROO ketogenic diet will be determined by participants achieving a ketogenic ratio (KR) value of > 1.5 on DHQ-III surveys or by being in nutritional ketosis in 80% of the ketone readings throughout the trial. KR value ≥ 1.5 is considered the minimum threshold to predict a ketogenic diet and will be calculated as follows: (0.9*grams fat + 0.46*grams protein) divided by (0.1*grams fat + 0.58*grams protein + grams total carbohydrate – grams total fiber). In the event the study intervention becomes too restrictive or difficult for the study participant to maintain, the study team has created diet adherence guidelines to give participants options to ease the burden of the study intervention and prevent study discontinuation (Table 2). One of the senior investigators, who has extensive experience conducting long-term, randomized controlled dietary intervention trials, was unblinded and responsible for monitoring and supporting participant adherence to the dietary protocols.

**Table 2:**
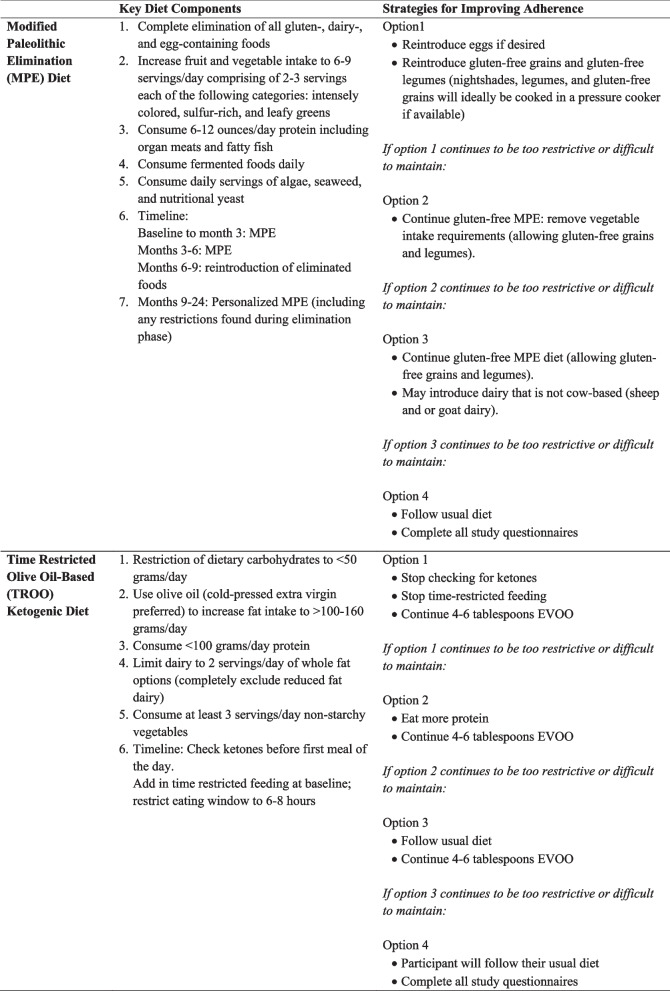
Intervention diets adherence guideline

### Relevant concomitant care permitted or prohibited during the trial {11d}

We ask the participants not to start any new supplements, over-the-counter medications, or new MS self-care interventions (e.g., sauna, cold plunges, yoga) during the study unless directed by their physician. In addition, participants are to report any concomitant medications including prescription medications, therapies for treating adverse events, MS relapses, over-the-counter medications, and dietary supplements.

### Provisions for post-trial care {30}

The participants randomized to usual care group will be offered the opportunity to elect to receive educational resources for one (but not both) of the intervention diets, MPE or TROO ketogenic, at their month 24 (end of study) visit.

### Outcomes {12}

#### Primary outcome

The primary outcome of this study is changes in mental and physical QoL scores over time in the intervention groups compared to the control group assessed using the MS Quality of life 54 (MSQoL-54) questionnaire, which is a validated 54-item questionnaire of MS-specific physical- and mental health-related QoL.

#### Secondary outcomes

Secondary outcomes include objective disease course (MRI brain imaging), ocular structure and function, perceived fatigue, mood, self-reported disability status, motor function, and cognitive function. Table 3 summarizes the primary and secondary outcomes and measures.

**Table 3:**
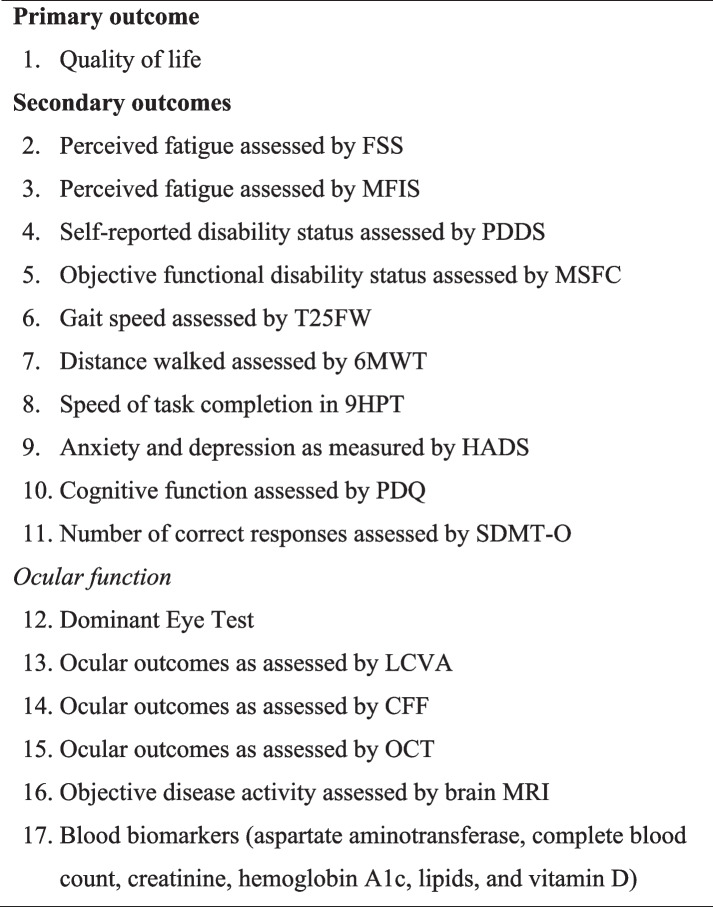
Study outcomes

#### Exploratory outcomes

Exploratory outcomes include identifying potential mechanisms via single voxel magnetic resonance spectroscopy (MRS) in frontal lobe white matter to identify brain metabolite changes over time.

### Participant timeline {13}

**Fig. 1:**
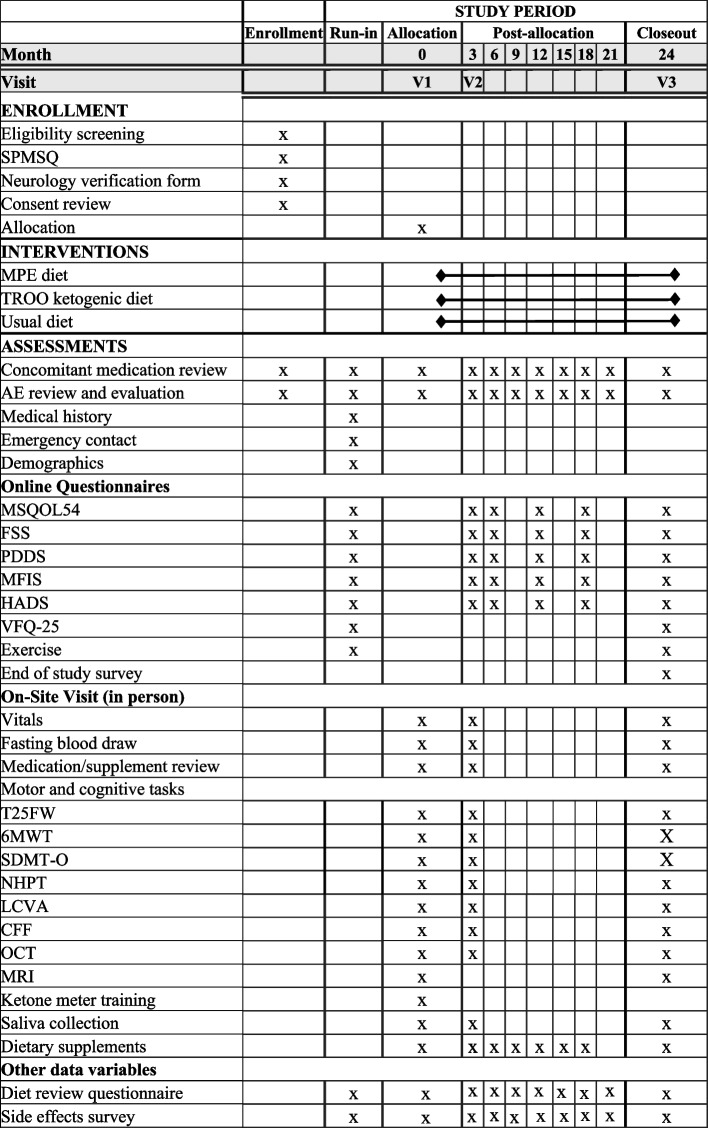
Standard Protocol Items: Recommendation for Interventional Trials (SPIRIT): the schedule of enrollment, interventions, and assessments

**Fig. 2:**
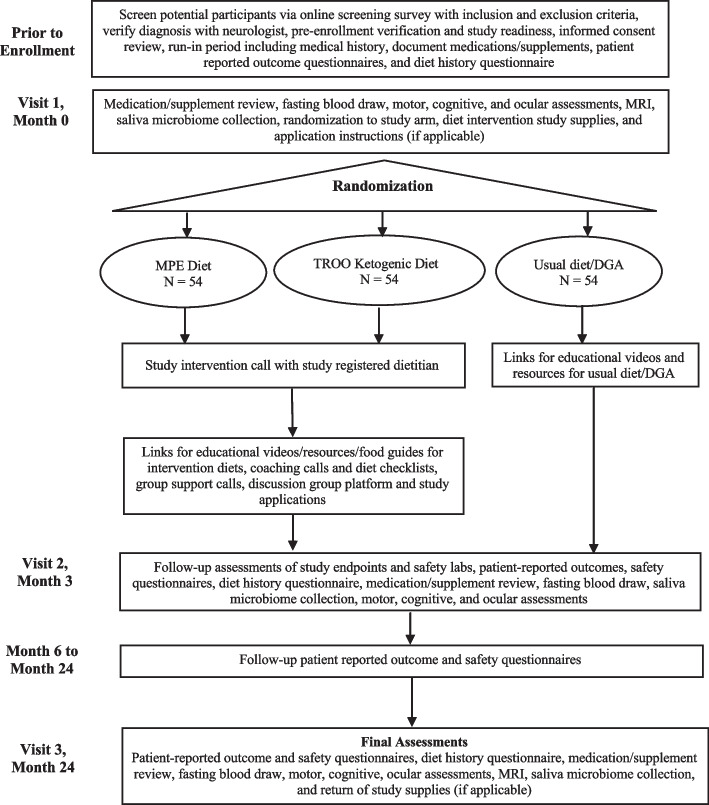
Efficacy of Diet on Quality of Life in Multiple Sclerosis (EDQ-MS) study flow diagram

The protocol was prepared according to the Standard Protocol Items: Recommendations for Interventional Trials (SPIRIT) guidelines [34] (Fig. 1; and Additional file 3: SPIRIT checklist). Figure 2 shows the EDQ-MS study flow diagram.

### Sample size {14}

Sample sizes required to achieve 80% power for testing between-treatment changes over time were calculated for each measure using summary statistics at baseline and 6-months from our previous randomized parallel arm trial comparing the LSF and MPE diets.

Using a group sample size of 36 and an alpha of 0.05, power and estimates for mean difference within and between groups are shown in Table 4. To ensure adequate sample size at the primary endpoint of the study, we will enroll enough participants to cover a 33% attrition rate in each group, giving a final target enrollment sample size of *n* = 54 per group.

**Table 4:**
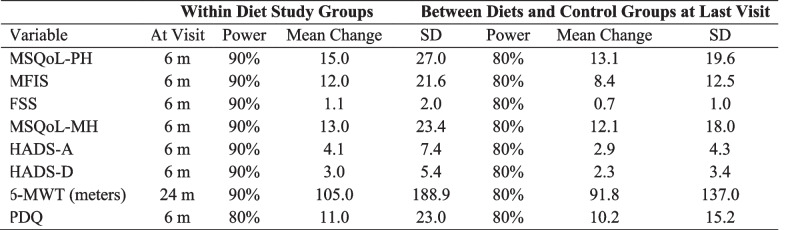
Power and sample size justification

### Recruitment {15}

The research team will post key study information on the National MS Society (NMSS) website, regional MS centers, the University of Iowa Hospital’s Neurology Clinic, terrywahls.com, Wahls Research Registry, social media advertisement, podcasts, posts, blogs, interviews, press releases, and other organizations to recruit study participants. Additionally, the study team will access the following study populations to recruit enough study participants to meet study recruitment goals: 1) send recruitment letters to neurologists at the University of Iowa and regional practices; 2) neurologists at the University of Iowa can invite their clinic patients; 3) mail posters and flyers/brochures to neurologists; 4) send emails to University of Iowa staff and students; 5) work with medical centers in Iowa to distribute flyers; 6) distribute brochures at professional and MS-related meetings; 7) post study information on NMSS website.

### Assignment of interventions: allocation

#### Sequence generation {16a}

Study participants will be randomized and evenly allocated at a 1:1:1 ratio to one of the following groups: MPE diet, or TROO ketogenic diet, or usual care control with DGA educational material, to remove bias from any staff performing study assessments responsible for the primary outcomes.

#### Concealment mechanism {16b}

The randomization table will be password-protected and accessible only to study RDNs and unblinded staff, ensuring it remains concealed from all blinded staff to uphold the integrity of the blinding process.

#### Implementation {16c}

Eligible participants will be informed of their assigned diet by the study RDNs during the baseline visit. Participants will be asked to discuss any diet-related concerns or questions with the RDNs or study coordinator to ensure that assessors and blinded staff remain unaware of their assignments.

### Assignment of interventions: blinding

#### Who will be blinded {17a}

Blinded staff include the PI, all co-investigators, and staff performing study visit assessments. Unblinded staff will include RDNs, study coordinator, and assigned research assistants working with data including, but not limited to, reviewing diet history questionnaires, Life Events (LE), study supplies, and adverse events (AE) reported by study participants. A member of the study team will inform study participants during the informed consent conversation that there will be blinded staff performing study assessments. In addition, statistical analysis of the study data will be performed independently by both a blinded and unblinded statistician.

#### Procedure for unblinding if needed {17b}

In the event of inadvertent unblinding by staff or study participant, the date and circumstance of the unblinding event will be documented. Following the unblinding, the research member of the team that was unblinded will not be allowed to perform any additional assessments on the unblinded study participant for the remainder of the study and a different assessor will be utilized at future study visits. In the case of a medical emergency, the study participant should inform all treating physicians what diet they were assigned for the study. Medical emergencies reported to the study team will be documented by unblinded staff and reported to the PI, Data and Safety Monitoring Board (DSMB), and IRB per Human Subject’s Office (HSO) reporting guidelines. In addition, once the final report has been reviewed by the DSMB, questions have been answered, and requested updates have been made, the DSMB will provide documentation that the PI and additional blinded staff may be provided with the randomized data.

## Data collection and management

### Plans for assessment and collection of outcomes {18a}

At baseline and every 6 months, participants will be sent links to complete web-based patient-reported outcomes assessments using validated questionnaires (Fig. 1). A custom, study-specific questionnaire will also be sent to participants every 3 months to monitor medical visits, perceived MS symptoms, life events affecting participation, and side effects of the dietary interventions. The following outcomes will be collected:

### Patient-reported outcomes

1. *MSQoL-54:* The MSQoL-54 questionnaire is a 54-item measure of physical- (35 items) and mental health (18 items)-related QoL, including 12 subscales. Higher scores indicate better QoL [35]. A 5-point change is considered clinically significant [36, 37]. In people with MS, the subscales have good internal consistency and test–retest reliability scores [35].
2. *Fatigue Severity Scale (FSS)*: The FSS is a nine-item questionnaire in which participants are asked to rate how fatigue affects their activities of daily life. Participants with a mean value of ≥ 4 are considered fatigued [38]. The FSS is a reliable and sensitive measure and has demonstrated internal consistency and discriminative properties among people with MS. Clinically meaningful reduction is defined as a decrease in score of at least 0.45 [39].
3. *Modified Fatigue Impact Scale (MFIS)*: The MFIS is a 21-item measure of fatigue’s effects on daily physical (9 items), cognitive (10 items), and psychosocial (2 items) function, where higher scores reflect a greater impact of fatigue [40]. Internal consistency is very good. Clinically meaningful reduction is defined as a decrease in score of at least 4.0 [39].
4. *Hospital Anxiety and Depression Scale (HADS):* The HADS is a 14-item self-report measure with two subscales assessing anxiety and depression [41]. A score of 8 or above on either subscale indicates clinically elevated symptoms of anxiety or depression. Clinically meaningful reduction is defined as a decrease in score of at least 1.5 for the Anxiety portion of HADs and 0.5 for the Depression portion of HADS [42]. The HADS has high sensitivity and specificity in people with MS [43–45].
5. *Patient Determined Disease Steps (PDDS)*: Disease status will be monitored with the PDDS, which was originally developed as a patient-reported surrogate to the Disease Steps [46]. The PDDS has nine levels of disease burden ranging between 0 (normal) to 8 (bedridden) [47] and can be converted into EDSS scores [48] as well as classifications of mild, moderate, or severe disability [49]. The PDDS has been validated to several measures of disability in MS [50]. A clinically meaningful change in PDDS is defined as a change in score of 0.99 points [51].

### In-person study visits

In-person study visits will take place at months 0, 3, and 24, and the following assessments will be performed.

Cognitive testing will be done using:

1. *Symbol Digit Modality Test—Oral (SDMT-O):* Participants must match as many symbols as possible to their corresponding numbers given in a key at the top of the page in 90 s [52]. The SDMT-O is the most commonly used evaluation of cognitive dysfunction in MS [53]. Clinically meaningful change is defined as an increase of at least 5.0 points for both mental and physical MSQoL-54 [54].

Assessments of motor function will be done with the following tests:

1. *Nine-hole peg test (NHPT)*: Participants place nine pegs (one at a time) into a block as quickly as possible, then remove them [55]. Two dominant hand trials are followed by two non-dominant hand trials; scores are the average time to complete the trials per hand. The NHPT has high test–retest reliability and discriminant validity in persons with MS vs. healthy controls [56–60]. A 20% NHPT score change is suggested as clinically meaningful [61, 62].
2. *6-min timed walk (6MTW):* Participants walk (not jog or run) laps around cones placed 100 feet apart for as many laps as they can complete in 6 min. During the timed 6 min, participants are allowed to rest if needed while a research team member keeps track of time and laps. At 6 min, participants are instructed to stop where they are and the distance from the last cone rounded is measured. The 6MTW has excellent test–retest reliability and discriminant validity between people with MS vs. healthy controls [63]. An increase of at least 6% for 6MTW is suggested as clinically meaningful [64].
3. *Timed 25-foot walk (T25FW)*: Participants walk 25 feet as quickly and safely as possible, then repeat the trial by walking back the same distance in the opposite direction. Participants may use assistive devices. The score is the average time of the two trials. The T25FW has demonstrated high test–retest reliability [60] and good concurrent validity [65]. A 20% change in walk time is suggested as clinically meaningful [62, 64].

Functional disability will be assessed by:

1. *MS Functional Composite (MSFC) (months 0, 3, and 24):* The MSFC is a validated measure of physical and cognitive function in MS that includes measures of upper extremity coordination (NHPT), gait speed (T25FW), and cognition (SDMT-O) [57, 66]. Raw NHPT, T25FW, and SDMT-O values will be converted to baseline-referenced z-scores and averaged to obtain an overall MSFC score [57]. The MSFC has good construct and concurrent validity; however, there is no recognized clinically significant change cut point [67]; though one study defined a clinically significant change as 0.5 MSFC points [68].

Ocular function will be assessed using:

1. *Dominant eye test (month 0):* This is a simple procedure used to determine which of a person’s eyes is dominant. The dominant eye provides a slightly greater degree of input to the visual part of the brain and relays information more accurately [69].
2. *Visual acuity:* The study participant will sit 6 feet from the chart, cover one eye with a handheld ophthalmic eye occluder (starting with testing right eye), and starting at the top, slowly read the chart out loud, starting with the biggest row of letters and moving down to smaller rows until letters are not seen clearly. The test is repeated for the other eye. If the participant does not reach 20/40 visual acuity, the trial will be repeated using a pinhole eye occluder.
3. *Contrast sensitivity:* The study participant will repeat the procedures of the visual acuity test for each eye with lighter charts at 2.50% and 1.25% contrast.
4. *Critical flicker fusion (CFF):* This is a validated measure to assess optic nerve function in the setting of MS. Improvement by at least 2 Hz is considered clinically meaningful change [70].
5. *Optical coherence tomography (OCT) (months 0 and 24):* This test measures the thickness of the retinal nerve fiber layer and the retinal ganglion cell layer. We will correlate thickness of these layers, CFF threshold, and low contrast visual acuity (LCVA) to assess changes in ocular measurements over the course of the study and between groups.

Disease activity will be assessed by:

*Brain Magnetic Resonance Imaging/Spectroscopy (MRI/S) (months 0 and 24)*: *MRI*: Two of the primary markers for MS progression on MRI images are lesion burden based on FLAIR hyperintensities [71–73] and changes in T1-weighted brain tissue volumes [72, 73].

1. *Magnetic resonance spectroscopy (MRS)*: The current study will include single voxel MRS in frontal lobe white matter to identify brain metabolite changes over time. The ^1^H MRS data will be analyzed using the LCModel [74]. Water suppressed and non-water suppressed data will be analyzed to generate absolute concentrations after performing phase and eddy current correction. Concentration measurements for N-acetyl aspartate (NAA), glutamate + glutamine (Glx), total creatine (Cr + PCr), total choline (tCho), myo-inositol (mI), and lactate will be obtained from the ^1^H data.

Nutritional safety of the dietary interventions will be monitored by:

1. *Blood measurements:* Blood will be collected at each in-person study visit. The UI Department of Pathology will analyze whole blood, serum or plasma for creatinine, aspartate aminotransferase, complete blood count, glucose, β-hydroxybutyrate, insulin, hemoglobin A1c, lipids, and vitamin D at months 0 and 24 visits. Hemoglobin Alc, lipids, and glucose will be analyzed at month 3 visits.

Exploratory analyses.

1. *Saliva biospecimens*: Specimens will be collected at months 0, 3, and 24 study visits and frozen for future microbiome analysis.
2. *Blood samples:* Samples will be collected at months 0, 3, and 24 study visits and frozen for future mechanistic analyses.

### Dietary assessment

Dietary assessment will be collected by asking study participants to complete the DHQ-III survey at months 0, 3, 12, and 24. Additionally, participants randomized to the MPE or TROO ketogenic diets will be instructed to record key study diet components each day using a study specific three-item questionnaire in the study-related (MyCap) application on their smart device. To decrease participant burden, completing MyCap is optional. The Healthy Eating Index (HEI), which is designed to determine how closely dietary intake mirrors the DGA, will be applied to the usual care control group to determine adherence to the DGA.

### Plans to promote participant retention and complete follow-up {18b}

The study team will use regular contact with study participants to identify times of stress that may result in participant dropout, and will use clearly defined protocol guidelines to allow participants to briefly modify dietary regimens and then quickly return to the study protocol to avoid attrition. Staff trained in Motivational Interviewing (MI) may have brief MI sessions to help uncover barriers that prevent participants from meeting goals. All study participants will receive retention messages from the study team which may include messages for birthdays, study anniversaries, holidays, visit and survey completion reminders, and check-ins via email, video message, and text message. Additional messages will be sent as needed to keep study participants feeling engaged and appreciated. Project Management Group (PMG) will hold weekly meetings to monitor participant engagement, proactively identify individuals at risk of dropout, and implement tailored strategies to enhance retention and ensure continued participation throughout the study. Study participants will be compensated for attending site visits and completing online surveys. $100 will be paid for months 0, 3, and 24 site visits following the completion of the study assessments and online surveys. $25 will be paid for the completion of online outcome surveys every 6 months between site visits.

### Data management {19}

Electronic data will be kept on a shared network folder managed by UIHC. The database will be password-protected with access limited to approved members of the study team. Subject data will be stored electronically in the Research Electric Data Capture (REDCap) platform [75]. Only IRB-approved research team members will have access to the REDCap data platform. Each team member will be granted access to the REDCap data system through a secure login. In the REDCap data platform, primary data is secured in the HCIS Pomerantz Data Center. Data backups are secured in the ITS Lindquist Data Center. Operating system security includes secure logins, data encryption at rest, remote system logging and configuration and change management.

### Confidentiality {27}

Each study participant’s contact information will be securely stored for internal use during the study. Study identification (ID) numbers will be used on data collection materials. Files that contain participant information will be kept in locked rooms. All electronic data will be kept on the UIHC Internal Medicine network folder. Network access will be limited to approved members of the study team. Each study team will access the folder through a secure login.

### Plans for collection, laboratory evaluation and storage of biological specimens for genetic or molecular analysis in this trial and future use {33}

Serum, plasma, and red blood cells (RBCs) will be frozen and stored for future use at the UI Tissue Procurement Core (TPC). The TPC has restricted physical access to the facilities, password-protected access control to informatics systems, encryption of data, de-identification of specimens and data, use of coded specimen identifiers. Samples will be stored in facilities with ID badge-controlled access.

### Statistical methods

#### Statistical methods for primary and secondary outcomes {20a}

Data for this study will be stored electronically using the REDCap platform at the University of Iowa and analyzed in SAS® 9.4. Baseline demographic and clinical measures collected will be summarized and tested for differences between study groups. Categorical measures are displayed as counts (percentages) and use Pearson’s chi-square or Fisher’s exact tests, depending on expected crosstab counts. Continuous measure distributions will be assessed using histograms. Normally distributed measures will be displayed as means (standard deviations) and compared using two-sample t-tests. Non-normally distributed measures will be displayed as medians (interquartile ranges) and compared using the Wilcoxon rank sum test.

The generalized linear mixed modeling (GLMM) [76] framework will be used to test the main and interacting effects of diet and time on the outcome measures of interest while accounting for repeated measures from each participant. The GLMM framework allows for non-normal distributions among the outcome measures. Other potentially important variables (age, sex, BMI, baseline smoking status, baseline alcohol use, walking assistance, years of MS, DMT use, baseline vitamin D, baseline 6-min walk distance) will be considered for inclusion in each model to assess their relationship with the outcome and to determine if they modify the estimates for the diet and time interaction. For each unique model (outcome and predictor set combination), Akaike information criterion (AIC) [77] will be obtained and utilized for model selection. Point estimates, 95% confidence intervals, and p-values of the diet mean changes in outcome measures over visits will be generated for each optimal model. For primary data analysis, data from all participants randomized at baseline will be included in an intention-to-treat analysis. A secondary per-protocol analysis will be used to examine the participants who complete the primary endpoint at 6 months for patient-reported outcomes or had clinical outcome assessments at 24 months. Hypothesis testing of the main effects in each optimal model (according to AIC) will use the Bonferroni correction for multiple comparisons between the three study treatment arms. All analyses will be conducted with two-sided tests (alpha = 0.05) using SAS software version 9.4 (SAS Institute Inc.).

#### Interim analyses {21b}

Interim analyses will be conducted for DSMB reporting but will not be conducted for presentation or publication.

### Methods for additional analyses (e.g. subgroup analyses) {20b}

A sensitivity analysis including only participants who were adherent to the intervention will be conducted as outlined below.

### Methods in analysis to handle protocol non-adherence and any statistical methods to handle missing data {20c}

A sensitivity analysis including only participants who were adherent to their assigned study diet will be conducted. Participants in the MPE diet group will be included in the sensitivity analysis if DHQ-III survey indicate that they consumed within 20% of recommendations for key intervention diet components (≥ 5 cup equivalent servings of total fruit and vegetables). TROO ketogenic participants will be included in the sensitivity analysis if they achieved a ketogenic ratio (KR) value of > 1.5 on DHQ-III surveys or if they are determined to be in nutritional ketosis in 80% of the ketone readings throughout the trial. KR value ≥ 1.5 is considered the minimum threshold to predict a ketogenic diet and will be calculated as follows: (0.9*grams fat + 0.46*grams protein) divided by (0.1*grams fat + 0.58*grams protein + grams total carbohydrate – grams total fiber). Control participants will be excluded from the sensitivity analysis if they have a significant change in HEI 2020 scores, defined as 10 points or more in either direction from baseline values. In all analyses, missing data will be modeled within the GLMM framework, which provides better power and reduced bias compared to other methods to handle missing data.

### Plans to give access to the full protocol, participant-level data and statistical code {31c}

Participant-level data and statistical code generated during the current study will be available from the corresponding author upon reasonable request up to five years after completion of the trial.

## Oversight and monitoring

### Composition of the coordinating center and trial steering committee {5d}da

Screening and recruitment are conducted under the supervision of the PMG, which includes the PI, study coordinator, and designated research staff, including research interns and RDs. The PMG meets weekly to review trial conduct and monitor overall progress. All other study-related procedures are implemented by trained members of the PI’s team, who maintain daily communication to ensure coordinated and efficient trial operations. The Steering Committee, comprising the PI, senior investigators, study coordinator, and biostatistician, provides scientific and strategic oversight of the trial.

### Composition of the data monitoring committee, its role and reporting structure {21a}

Safety oversight will be under the direction of a Data Safety and Monitoring Board (DSMB) composed of individuals with the appropriate expertise, including physicians in the UI Departments of Neurology and Internal Medicine, and the UI College of Public Health. Members of the DSMB are independent from the study conduct and free of conflict of interest.

### Adverse event (AE) reporting and harms {22}

Possible AEs include: MS relapse, including use of corticosteroids; exacerbation of MS symptoms (pseudo-relapse), no corticosteroids; gastrointestinal (GI) symptoms/side effects; infection (e.g., COVID-19, upper respiratory, urinary tract); non-GI related/other; abnormal lab value(s).

A serious AE is any adverse experience (associated with the study intervention) that results in any of the following outcomes: 1) death; 2) life-threatening adverse drug experience; 3) inpatient hospitalization or prolongation of existing hospitalization; 4) a persistent or significant disability/incapacity; 5) a congenital anomaly/congenital disability; 6) important medical events that may not result in death, be life-threatening or require hospitalization but may be considered a serious adverse drug event when, based upon appropriate medical judgment, may jeopardize the participant and may require medical or surgical intervention to prevent one of the outcomes listed above.

### Frequency and plans for auditing trial conduct {23}

The DSMB will meet every six months in joint meetings with the Steering Committee to review cumulative safety and efficacy data from each study arm. The study team will also prepare and submit annual progress reports to the DSMB, summarizing enrollment status, adverse events, protocol deviations, and other relevant metrics. Following each DSMB meeting, the board will provide its recommendations and feedback directly to the PI, who will determine appropriate action in consultation with the Steering Committee. In addition to these formal oversight procedures, the PMG, comprising the PI, study coordinator, senior investigators, RDs, and research staff, meet weekly to monitor ongoing trial operations, address logistical or compliance issues, and ensure that the trial is progressing according to the established timeline and regulatory requirements.

### Plans for communicating important protocol amendments to relevant parties (e.g. trial participants, ethical committees) {25}

All protocol amendments will first be reviewed and approved by the Steering Committee and submitted to the IRB for ethical approval. Following IRB approval, the study funder will be notified. The PI will then communicate the approved amendments to all participating sites and study personnel. A copy of the revised protocol will be distributed and filed in the Investigator Site File at each site. Any deviations from the approved protocol will be fully documented using a breach report form, in accordance with institutional and regulatory guidelines. Additionally, the updated protocol will be registered in the appropriate clinical trial registry to ensure transparency and public access to the most current version of the trial design.

### Dissemination plans {31a}

We will disseminate our study results via peer-reviewed journals, abstract presentation at conferences, trial registers, and social media platforms.

### Patient and public involvement

The PI has MS, and we acknowledge the importance of patient-centered research. The PI holds annual meetings with Wahls Team donors, many of whom are individuals living with MS, to discuss ongoing research and gather their input on research priorities. The study design and protocol was developed by the investigative team in alignment with feedback obtained from study participants from prior pilot trials [78], clinical priorities and current research gaps in MS care. We recognize the value of incorporating patient perspectives into study design and implementation. As such we are obtaining feedback from participants in end of study surveys and will use the feedback to inform future research. Additionally, at the conclusion of the trial, and following the publication of primary outcome results in peer-reviewed journals, the PI will share the study findings through multiple dissemination channels, including MS-focused and wellness-related podcasts, YouTube interviews, and free educational webinars. These efforts aim to ensure the findings are widely accessible and relevant to individuals with MS, their families, and healthcare providers, including neurologists, primary care teams, integrative medicine practitioners, and RDs.

## Discussion

There is growing evidence that non-pharmacological interventions, particularly dietary interventions, are effective means to manage MS disease progression and symptom severity. Recently, two meta-analyses of randomized dietary intervention trials found a significant impact of dietary interventions on fatigue and QoL among people with MS [8, 79]. One of these meta-analyses [8] resulted in members of the Nutrition Workgroup of the National MS Society recommending a healthy diet as an adjunct therapy for people with MS [80]. Furthermore, a systematic evidence review, conducted by the National Institute for Health and Care Excellence (NICE) to assess the efficacy of non-pharmacological interventions, including diet, in the management of fatigue, found consensus on incorporating diet as a factor in tailored fatigue management recommendations, and underscored the significance of a healthy diet’s impact on overall health in MS [81].

Given the significant knowledge gap in specific dietary recommendations as part of MS care, need for better evidence, and patient demand for evidence-based dietary recommendations, randomized controlled trials with larger sample sizes, longer follow-up, and robust methodology are urgently needed to bridge the knowledge gaps in efficacy as well as the mechanisms by which diet impacts outcomes. Thus, this study protocol describes the design of a randomized, single-blind, controlled design trial, aiming to evaluate the efficacy of incorporating dietary guidance within MS care for improving long-term QoL compared to usual care. Based on the preliminary data obtained from previously conducted trials by our team using the same dietary approaches [22, 23, 82], we expect to see positive short- and long-term benefits on QoL (the primary outcome measure), fatigue, and cognition; however, we have not evaluated MRI in our previous studies and thus have no ability to predict these outcomes. In addition, we expect that these diets will be safe to follow long-term.

In comparison to previous dietary studies in the MS field, our approach offers several distinct advantages. First, brain MRI will be conducted at baseline and after 24 months following the diets, to monitor the number and volume of enhancing lesions and brain volume over time. We hypothesize that the intervention groups will have a significantly lower number of FLAIR-evident lesions, and reduced annualized brain volume loss compared to controls at 24 months. Furthermore, the proposed study includes a usual care control group, larger sample size (*N* = 162), and longer duration of follow-up (24 months), that not only improves scientific rigor but permits evaluation of long-term efficacy of the two dietary strategies in improving QoL and reducing fatigue. Finally, we propose to identify potential mechanisms, initially by including MRS in frontal lobe white matter to identify brain metabolite changes over time. Additionally, collecting blood and saliva samples will enable us to conduct further mechanistic analyses.

This study has major potential to critically evaluate the effects of specialized diets on QoL in persons with MS, thereby benefiting the MS community by providing rigorous evidence related to this preliminary hypothesis. The results from this study could help clinicians provide clearer dietary guidance to patients to potentially improve QoL.

### Trial status

This study protocol was approved on December 16, 2021, and this manuscript details the protocol in the original version. The first participant was enrolled on April 13, 2022, and recruitment was completed July 22, 2024. All study activities are anticipated to be completed by September 1, 2026.

## Supplementary Information

Supplementary Material 1

Supplementary Material 2

Supplementary Material 3

Supplementary Material 4

## Data Availability

Data supporting the findings of this study are available from the corresponding author, TLW, upon reasonable request.

## Abbreviations

6MTW: 6-Minute Timed Walk
AE: Adverse Event
CFF: Critical Flicker Fusion
DGA: Dietary Guidelines for Americans
DSMB: Data Safety Monitoring Board
eCRF: Electronic Case Report Forms
EDQ: Efficacy of Diet on Quality of Life
FLAIR: Fluid Attenuated Inversion Recovery
FSS: Fatigue Severity Scale
HADS: Hospital Anxiety and Depression Scale
IRB: Institutional Review Board
ITT: Intention-To-Treat
LCVA: Low Contrast Visual Acuity
MFIS: Modified Fatigue Impact Scale
MI: Motivational Interviewing
MPE: Modified Paleo Elimination
MRI: Magnetic Resonance Imaging
MS: Multiple Sclerosis
MSQoL54: MS Quality of life 54
NHPT: Nine Hole Peg Test
OCT: Optical Coherence Tomography
PDDS: Patient Determined Disease Steps
PI: Principal Investigator
QoL: Quality of Life
RDN: Registered Dietitian Nutritionist
RRMS: Relapsing-Remitting Multiple Sclerosis
SAE: Serious Adverse Event
SCT: Social Cognitive Theory
SDMT-O: Symbol Digit Modalities Test – Oral
SPMSQ: Short Portable Mental Status Questionnaire
T25FW: Timed 25-Foot Walk
TPC: Tissue Procurement Core
TROO: Time Restricted Olive Oil-based
USDA: United States Department of Agriculture
