# Supplementary material for: Efficacy of Diet on Quality of life in Multiple Sclerosis (EDQ-MS): a study protocol for a randomized controlled clinical trial
Source: Trials. 2025 Oct 27;26:437. doi: 10.1186/s13063-025-09157-2 (PMC12557952; doi:10.1186/s13063-025-09157-2)
Supplement: Supplementary file 1 — Supplementary Material 1 [file 13063_2025_9157_MOESM1_ESM.pdf]

FOR IRB USE ONLY
APPROVED BY: IRB-01
IRB ID #: 202104639
APPROVAL DATE: 11/28/23

# INFORMED CONSENT DOCUMENT

Project Title: **Efficacy of Diet on Quality of Life in Multiple Sclerosis**
Project Subtitle: **EDQ-MS**

**Principal Investigator:** Terry Wahls MD, IFMCP
**Co-Principal Investigator:** Linda Snetselaar PhD, RD, LD

**Research Team Contact:** Mary Ehlinger, Clinical Research Coordinator can be reached at **319-384-5002.**

This consent form describes the research study to help you decide if you want to participate. This form provides important information about what you will be asked to do during the study, about the risks and benefits of the study, and about your rights as a research subject.

- If you have any questions about or do not understand something in this form, you should ask the research team for more information.
- You should discuss your participation with anyone you choose such as family or friends.
- Do not agree to participate in this study unless the research team has answered your questions and you decide that you want to be part of this study.

## WHAT IS THE PURPOSE OF THIS STUDY?

This is a research study. We are inviting you to participate in this research study because you have been diagnosed with relapsing remitting multiple sclerosis (MS) and have expressed interest in a dietary approach to treating your disease. After you review this informed consent document, we will schedule a Zoom meeting or phone call with you, to go through this document section by section, answer any questions you have, and send you an electronic survey and form asking questions to make sure you understand what is being asked and wish to participate in the study. After this is completed, we will ask that you sign the informed document to signify your desire to participate in the study.

The purpose of this research study is to compare the effect of three diets in patients with relapsing remitting multiple sclerosis. Currently, we don't know which diet is best. We would like to evaluate if adopting a Modified Paleo Elimination (MPE) diet, a Time Restricted Olive Oil-based (TROO) Ketogenic diet, or your usual diet with educational materials will improve quality of life and reduce fatigue in study participants diagnosed with multiple sclerosis. Fatigue is a disabling symptom that can significantly interfere with the ability for people to effectively function at home and work. You will be randomized 1:1:1; which means that you will have an equal chance of being assigned to any of the 3 above mentioned diets; therefore, **we need participants who are willing to follow any of the 3 diets and make changes in the foods they eat.** We will use videos and Zoom calls to teach you how to adopt the TROO ketogenic diet or the MPE or will provide you links to educational PDFs and videos for additional information to use with your usual diet.

## HOW MANY PEOPLE WILL PARTICIPATE?

A total of about 5,000 people with multiple sclerosis will take part in this study conducted by investigators at the University of Iowa. Approximately, 150 people will be enrolled in the study for participation. Additionally, there may be up to 4500 participants that will agree to be a part of our research registry when screening for this study. Participants that qualify for the study during the

screening phase will be asked for permission to contact their neurologist and/or be enrolled in this study.

## HOW LONG WILL I BE IN THIS STUDY?

If you agree to participate in the study your involvement will last for approximately 2 years. Three site visits will be held at the University of Iowa Hospitals and Clinics. The first site visit will be your baseline visit, you will return to the site at Month 3, and again at Month 24. Each site visit will last approximately 3 to 6 hours. Between site visits, online survey invitations will be sent to be completed at your home every 3 months (0, 3, 6, 9, 12, 15, 18, 21, and 24).

After Month 24, there is an option to continue involvement for another 4 years in a long-term follow-up study. This optional follow-up period will consist of online surveys only (no site visits). The questionnaires will be sent to you 1 month after your last site visit (Month 24) and every 6 months for the additional 4 years, for a total of 6 years (72 months). The surveys will ask about changes in your diet, if you experience a relapse, if you change your MS medications, and how you are feeling. You will **NOT** be expected to maintain the diet you were randomized to for the additional 4 years in the long-term follow-up study.

**Please initial below to indicate your preference:**

__________ **Yes, I agree to participate in the 4-year long-term follow-up study.**

__________ **No, I DO NOT agree to participate in the 4-year long-term follow-up study.**

## WHAT WILL HAPPEN DURING THIS STUDY?

During this study you be given access to instructional videos and several resources to provide education for 1 of 3 study diets: Modified Paleo Elimination (MPE) Diet, Time Restricted Olive Oil-based (TROO) Ketogenic diet, or educational materials that you may implement with your usual diet. We will send your doctor a letter to inform him/her that you are interested in participating in a research study focusing on diet and MS and ask that they confirm your RRMS diagnosis. This will also give us permission to communicate with him/her throughout the duration of the study.

We will ask you to report to us any new symptoms, changes in health, or changes in medications (i.e., new, or worsening illness) while participating in the study. We will ask for information and request medical records if needed regarding these events to document them appropriately.

**Run-In Period.** This is a 14-day period after you have consented for the study to collect important information before you are randomized to one of the three diets. This period may take up to 14 days, however, if all tasks are completed sooner a site visit may be scheduled. This will include initial study surveys and an interview with the study research team. You will meet with the study staff via Zoom meeting to collect your medical history including your family health issues and symptoms. This will also include functional medicine questions about your health from infancy to the current time.

**Randomization.** To participate in this study, you must agree to follow any of the three diets that we are studying for approximately 2 years. The three diets include Modified Paleo Elimination (MPE) diet, Time Restricted Olive Oil-based (TROO) Ketogenic diet, and your usual diet with educational materials. Below is a table of key components for each of the diets we are comparing:

| **Modified Paleo Elimination (MPE) Diet** | **Time Restricted Olive Oil-based (TROO) Ketogenic Diet** | |
|---|---|---|
| 1. Elimination of all gluten-, dairy-, and egg-containing foods.<br>2. High fruit and vegetable intake to 6-9 servings/day comprising of 2-3 servings each of the following categories: colored, sulfur-rich, and leafy greens.<br>3. Eat 6-12 ounces/day protein including organ meats and fatty fish.<br>4. Eat fermented foods daily.<br>5. Eat daily servings of algae, seaweed, and nutritional yeast. | 1. Limit of dietary carbohydrates to < 50 grams/day.<br>2. Use olive oil (cold-pressed extra virgin preferred) to increase fat intake to >160 grams/day.<br>3. Eat <100 grams/day protein.<br>4. Limit dairy to 2 servings/day of whole fat options (completely exclude reduced fat dairy).<br>5. Eat at least 3 servings/day non-starchy vegetables. | **Usual Diet w/ additional Education Information provided monthly** |

**Blood collection.** To participate in this study, you must agree to have your blood drawn to monitor potential changes in your nutritional biomarkers (blood tests of your nutritional status). Any changes in the biomarkers will be followed and compared to the presence or absence of changes in your physical condition, thinking ability and mood over time. Approximately 9 teaspoons of blood will be drawn at each visit. This blood draw, along with the gathering of other vitals (heart rate, blood pressure, etc.), will take 15-25 minutes to complete. Your blood samples will be labeled with your study ID and transported immediately to a laboratory in the UIHC hospital for analysis or frozen and stored in the Tissue Procurement Center for future analysis. The future analysis of your blood samples may be done at the University of Iowa or other future IRB approved medical centers and team members. **Blood is a very important component to this study. If we are unable to draw blood at a visit, or if the blood was not properly processed by lab staff, we will ask that you have your most recent laboratory results sent to the study team. If you do not feel comfortable having blood drawn or are unable to have blood drawn, the study team can end your participation in the study. The research study <u>will not</u> include whole genome sequencing as part of this research study.**

**Physical Motor Tasks.** You will complete the MS Functional composite. This test consists of a 25-foot walk test which is completed twice (once in each direction) and a test of hand coordination that is completed with both the right and left hands. You will also complete a symbol digit task that asks you to substitute a number for meaningless geometric shapes based on a key that assigns a specific number to each shape. You may rest after the tests. Then you will complete the 6-minute walk test. You may rest after the test. These tests will be conducted at each site visit. To prevent increased fatigue prior to your motor tests, we can transport you in a wheelchair to the various parts of the visit that require us to leave the study office. This includes going to the blood draw room and to the hallway where we will perform the 6-minute walk test. **Please do not tell the study staff conducting these measurements anything about being on a study diet or taking study supplements; the staff conducting these tests must not know what intervention you are assigned.**

**Surveys.** Throughout this study you will be asked to complete several different surveys. These questionnaires include information about your demographic data, emergency contact, physician's contact information, doctor's appointments, life events, the foods you are eating, medical symptoms, exercise, sleep, mood, stress, medications, supplement intake, and adverse childhood events. While we

ask that you answer all questions completely and to the best of your ability, you are free to skip questions that you would prefer not to answer. All questionnaires may be completed at home or on your smartphone or tablet device with a secured website (REDCap) during the run-in period and every 3 months after your first study visit. You will receive an email request from REDCap asking you to complete questionnaires online. You will have approximately 5 days to complete these surveys. The email will contain a link to the surveys. The questionnaires may take up to 2 hours to complete.

The questionnaire regarding adverse childhood events will ask sensitive questions that may cause you to feel uncomfortable or be difficult to complete. We ask that you complete this questionnaire one time at the beginning of the study, and it is optional. You can choose NOT to complete any, 1, or more of the questions. We are asking you to complete this questionnaire because in previous studies, several participants have indicated childhood adverse events during a functional medicine interview. We would like to obtain preliminary data to see if there is a higher incidence of these types of events in people diagnosed with MS.

**Please initial below to indicate your preference:**

__________ **Yes, I agree to take the adverse childhood events questionnaire.**

__________ **No, I DO NOT agree to take the adverse childhood events questionnaire.**

**Vital Signs.** At all site visits we will measure your vital signs including weight, pulse (heart rate), temperature, waist circumference, and blood pressure. At visit 1, we will also measure your height to calculate your body mass index (BMI, a measure of your weight and height).

**Dietary Intake Assessment.** Following your consent call, you will be asked to complete a web-based diet history questionnaire. You will be given an unidentifiable login and password with a unique survey link for each survey request. You will be asked to complete this questionnaire before the first site visit, prior to the month 3 visit, at month 12, and prior to the last site visit at month 24. At visit 1, you will be randomized to your study diet.

NOTE -- You should **ONLY** agree to participate in this study if you are confident that **1) you are willing to be randomized to any of the 3 diets and can adopt and sustain the study diet to which you are randomized and 2) your family is willing to support you in following the study diet.**

You may find it helpful to remove from the house foods that are not recommended for the study diet to which you are randomized**.** The foods that will be excluded from the study diet to which you are randomized may include foods that you and your family currently enjoy such as convenient meal options and snacks, food with added sugar, dairy, eggs, gluten, or grain-based products, and you may be encouraged to eat foods that are unfamiliar to you. You will be given resources and websites to help you adopt the relevant assigned study diet.

If you’re assigned the Modified Paleo Elimination (MPE) diet or Time Restricted Olive Oil-based (TROO) Ketogenic diet, you will learn how to record a 3-question daily diet checklist to fill out for the duration of the study. This will require a free application to be downloaded to your smartphone or tablet device and will take less than 30 seconds to complete.

In addition, if you are assigned to the Time Restricted Olive Oil-based (TROO) Ketogenic diet group, you will also need to download a second free application to your phone. The applications needed include MyCap and Keto-Mojo and can be found in the Android or Apple app stores. The companies that make these applications will be able to see your activity and account setup data. They will not have access to your medical records. You may choose a login name other than your real name.

Following visit 1, you will receive access to videos and/or resources explaining your assigned study diet. If you're assigned the Modified Paleo Elimination (MPE) diet or Time Restricted Olive Oil-based (TROO) Ketogenic diet, there will be an initial Zoom Meeting video call to answer questions and help with instruction prior to beginning the study diet. Monthly group Zoom video support meetings and an online "Canvas classroom" support will also be provided throughout the duration of the study to participants assigned the Modified Paleo Elimination (MPE) diet or Time Restricted Olive Oil-based (TROO) Ketogenic diet.

An additional resource developed for this study includes a recipe and meal planning application if you are assigned the Modified Paleo Elimination (MPE) diet or Time Restricted Olive Oil-based (TROO) Ketogenic diet. You will only be given access to recipes that are consistent with the key components of the study diet you are assigned. This application is free to download and is NOT required for participation in the study. The purpose of the EDQ-MS application is to provide assistance, resources, and support during your study participation. A non-identifiable email address will be created by the research team to protect your identity and an invitation will be sent to this address for approval, set-up, and access to the application.

**Saliva Collection.** One aspect of this study is to analyze what bacteria and other microbes are living in your gut. At all site visits, we will collect a saliva sample by spitting a small amount of saliva in a collection tube. You will be asked not to eat, drink, smoke, or chew gum for 30 minutes before giving their sample. We prefer that you take no new over the counter vitamins or supplements for one week prior to collecting the saliva specimen.

**Zoom Meetings.** You will be scheduled Zoom Meetings with the study team at pre-enrollment to discuss eligibility and willingness to conduct study procedures, review the consent form in detail, discuss medical history, and review medications and supplements. If assigned the Modified Paleo Elimination (MPE) diet or Time Restricted Olive Oil-based (TROO) Ketogenic diet, you will also meet with the study dietitian to review your study diet. You will be given times for Zoom Meeting calls over the course of the first month with the study dietitian and then monthly calls with the study team and other study participants. You may contact the dietitian and study team via phone, email, or Canvas classroom at any time to receive support in adopting and maintaining the assigned study diet.

**Audio Recording.** One aspect of this study involves making audio recordings of your enrollment, consent, dietary training with the dietitian, group monthly phone calls, and in-person diet counseling sessions at the final visit. They will be reviewed by the dietitian and identified study team members for quality control and training purposes. The recordings are optional. You will be notified at the beginning of the Zoom meeting when the recording will begin. You may cancel this consent at any time during the conversation by asking the study team or dietitian to stop the recording.

**Please initial below to indicate your preference:**

**___________ Yes, I give you permission to make audio recording of me during this study.**

**___________ No, I do not give you permission to make audio recording of me during this study.**

**Supplements and medications.** If you are assigned the Modified Paleo Elimination (MPE) diet or Time Restricted Olive Oil-based (TROO) Ketogenic diet, we will dispense to you specific vitamin and mineral supplements to take during the study (2 capsules of BodyBio Balance Oil (1400 mg linoleic acid/350 mg linolenic acid), 1 capsule Kirunal Fish Oil (315 mg eicosapentanoic acid/ 105 mg docosahaenoic acid) and 2 capsules of BodyBio PC (1300 mg phosphatidyl choline). You will be asked to begin taking the study supplements one supplement at a time and then adding another new one every 4th day. For example, if you were to start the first supplement on day 2 following your Month 0 / baseline visit, the remainder of the supplements would be added on days 6 and 10. This will allow time to determine if you experience any side effects. If side effects are noted, stop the supplement, and notify study staff immediately. Besides these three supplements, you should not begin taking any new supplements during the study unless prescribed by your physician. The target serum vitamin D levels are 50 - 80 ng/ml. Vitamin D values will be checked at Month 0 and Month 24. If you are not in a healthy range of Vitamin D your neurologist and primary care physician will be notified. At your Month 0 / baseline site visit, we ask that you bring all prescription medications and dietary supplements you are taking.

**Eye and Associated Measurements.** Another aspect of this study is to monitor how effectively the brain, central nervous system and eye system can repair itself if given a more optimal environment. To study this, we are going to perform eye tests (outlined below) at each site visit. These measurements have minimal risk and provide an effective means to monitor disease progression. You will need to bring your glasses for both reading and distance for these tasks (if applicable). With your permission, your eye doctor will be contacted to retrieve prescription information and/or old test results. The results from these measurements will be used for research purposes only and will be evaluated for changes over time. Therefore, the results will not be analyzed until after the end of the study. After analysis, if there are unexpected findings, you will be notified so they can be shared with your treating physician.

**Dominant Eye Test.** This test will take less than a minute to complete and will only be conducted at the Month 0 / Baseline visit. While both your eyes are open you will point with your index finger exactly at the letter projected on the screen in front of you. The examiner will cover your eyes one at a time to check which eye you see your finger pointed directly at the letter.

**Critical Flicker Fusion Test (CFF).** This test will take approximately 10 minutes and will be conducted at all site visits. You will look in a dark box containing a red light. For the first test, this light will be flickering, and the speed of the flickering will increase. Your job will be to indicate when the light no longer appears to be flickering by pushing a button. The test will then be repeated, except this time the red light will be continuous. Your job will be to indicate when the light begins to flicker by pressing a button. The test may be repeated for each eye three times to obtain accurate results.

**Optical Coherence Tomography (OCT).** This is a non-invasive scan of the eye which will use light beams to produce a picture of your retina. You will undergo OCT scanning of your eyes that might take up to 30 minutes. This test will be conducted at the Month 0 / Baseline and Month 24 / End of Study

visits. If the study team is unable to capture a scan at Month 0, another attempt will be made at Month 3. With your permission, the examiner may use artificial tears to improve your comfort and scan quality. **NO** dilating drops will be given. You will be asked to focus on the small green fixation target without blinking while the machine focuses on your retina. The examiner will then begin the scan. Blinking during the scan is allowed as it will not affect the picture taken, however, blinking can make the procedure take longer. This will be done for each eye. You will be allowed to relax between scans.

**Visual Acuity and Contrast Sensitivity Testing.** This test is like the test you perform in eye clinics. You will be seated 2 meters in front of a chart. The letter size will vary in terms of size and then darkness or contrast. Your job will be to indicate the name of letter until you are unable. Each eye will be tested individually. The test will be repeated with a chart using a progressively lighter shade of gray. This test will take approximately 7-10 minutes and will be conducted at all site visits.

**MRI Scans.** To participate in this study, you must be able to undergo MRI imaging of your brain. We will ask you to have 2 non-contrast MRIs for this study 24 months apart (Month 0 / Baseline and Month 24 / End of Study) during you site visits.

An MRI scanner takes pictures of the inside of your body by sending out a magnetic field and radio waves. Because the MRI scanner contains a very strong magnet, you may not be able to have the MRI if you have certain kinds of metal in your body (for example, a heart pacemaker, a metal plate, and certain types of heart valves or brain aneurysm clips). Someone will ask you questions about this before you have the MRI.

The MRI scanner is a large machine that contains a hollow tube. You will be asked to lie on your back on a special table that slides into the tube. The sides of the tube will be close to your body and the scanner makes a loud hammering noise while you are inside. You will be able to talk to people in the room through a speaker system. We will monitor you closely while you are inside the scanner.

**<u>HOW TO PREPARE FOR STUDY VISITS.</u>**

**Fasting**. We ask that you **fast for 8 hours prior to each site visit**, which means no food or beverages may be consumed other than water. Please ensure that you are staying well hydrated 1-2 days prior to your visit. **<u>Please do drink water and take your prescribed medications while you are fasting.</u>** Drawing the blood will be easier if you are well hydrated. You may want to bring a snack to eat after the blood draw is complete. You may also purchase food in the hospital cafeteria.

**Prescription Medications, Dietary Supplements, Over-the-Counter Medications**. We request that you bring the bottles of ALL your prescription and over-the-counter medications and supplements you are taking to the Month 0 / Baseline visit. Please bring the bottles of any new prescriptions to the Month 3 and Month 24 / End of Study visit. We ask that you not start any new supplements or over-the-counter medications during the study unless directed by your physician.

**<u>STUDY VISITS.</u>**

**Month 0 / Baseline Visit will take approximately 4-6 hours to complete.** The first visit will occur following electronically signing this consent via REDCap E-consent and a 14-day run-in period. We will ask you to fast for 8 hours (stop eating and drinking all beverages, except water) after midnight before your visit. We will request you start drinking water prior to the site visit to make it easier for staff members to draw your blood. We will measure your vital signs (weight, pulse, temperature, blood

pressure), height, waist circumference, and calculate your body mass index (BMI, a measure of your weight and height).

We will review your supplements and medications that were recorded while documenting your medical history. You will have blood drawn and a saliva sample collected. You will complete physical motor tasks, a cognitive test, eye function tests and an MRI. MRI's will be scheduled according to available times during your scheduled visit.

You will be randomized (or assigned) your study diet. If you are assigned the Modified Paleo Elimination (MPE) diet or Time Restricted Olive Oil-based (TROO) Ketogenic diet you will receive training on how to keep a short daily electronic checklist. If you are assigned the Time Restricted Olive Oil-based (TROO) Ketogenic diet you will also receive training for ketone monitoring. You will be given access to instructional videos and/or additional resources specific to your assigned study diet. If you are assigned to the Modified Paleo Elimination (MPE) diet or Time Restricted Olive Oil-based (TROO) Ketogenic diet you will schedule Zoom meetings with the study registered dietitian. You will record daily food logs into your smartphone application, and you will be asked to watch videos and review resources to make sure that you are comfortable following the study diet. You will be given a study schedule outlining the dates for future group calls, online survey requests every 3 months and target dates for your Month 3 and Month 24 site visits as well as resources with contact information for the study team. If you are assigned your usual diet with educational materials, you will receive texts messages and / or emails approximately once a month with resource links about your diet and information about recent multiple sclerosis-related research.

**Month 3 will take approximately 2-3 hours.** We will ask you to fast for 8 hours (stop eating and drinking all beverages, except water) after midnight before your visit. When you arrive at the PIC, we will request you start drinking water prior to the visit to make it easier for staff members to draw your blood. Your weight, temperature, pulse, blood pressure, and waist circumference will be recorded. You will have your blood drawn and a saliva sample collected. We will review the medications and supplements you are taking and ask about any changes to your health. You will complete physical motor tasks, and eye tests (visual acuity, contrast sensitivity, and critical flicker fusion). You will meet with the study dietitian to review your successes and challenges and so you can ask questions and receive support in continuing to follow the diet.

**Month 24 Visit will take approximately 4-6 hours to complete.** We will ask you to fast for 8 hours (stop eating and drinking all beverages, except water) after midnight before your visit. When you arrive at the PIC, we will request you start drinking water prior to the visit to make it easier for staff members to draw your blood. Your weight, temperature, pulse, blood pressure, and waist circumference will be recorded. You will have your blood drawn and a saliva sample collected. We will review the medications and supplements you are taking and ask about any changes to your health. You will complete physical motor tasks, eye tests (visual acuity, contrast sensitivity, and critical flicker fusion) and an MRI. MRI's will be scheduled according to available times during your scheduled visit.

In addition, we will review your end of study survey to discuss feedback on how our future studies can improve.

**Blood/Saliva Data Storage for Future Use.** As part of this study, we are obtaining blood and saliva

samples from you. We would like to study your blood and saliva samples in the future, after this study is over. Your sample, information, and/or data may be placed in a central repository or other national repositories sponsored by the National Institutes of Health or other Federal agencies. If this happens, it will be stripped of identifiers (such as name, date of birth, address, etc.). Other qualified researchers who obtain proper permission may gain access to your sample and/or data for use in approved research studies that may or may not be related to in the purpose of this study.

Blood cells removed from the blood samples will be used to make a cell line and DNA or conduct whole genome sequencing. Cell lines are produced by growing blood cells in a laboratory and allow us to have a source of the DNA without having to redraw your blood. These blood cells can be stored for decades or more. The DNA, blood, and saliva data will be made available to researchers trying to learn more about the cause of diseases.

Each of the cells in your body contains DNA. DNA is the instruction manual that determines your appearance in things like eye color or how tall you can be. Your DNA may also lead to higher or lower risk of certain diseases. Your environment will also determine some of your disease risk.

Your DNA is a string of four building blocks, called “bases.” These bases are represented by the letters G, A, T, and C. There are billions of these letters strung together in every human’s DNA and they are arranged in packages like words. Each of these “words” have specific jobs in the body. Most of the time the letters are the same in everyone. But about 1% of the population might have an “A” where someone else has a “G.” This difference can explain why some people have blue eyes and others brown eyes, or why some have a high risk for a certain cancer and others a low risk. All these letters come together to create your “genome sequence”, a kind of book of your genetics. It now possible to read off each of these letters and read your complete genome sequence. Your DNA sequence is unique to you. You inherit your DNA in almost equal parts from each of your parents. In very rare cases, your genome can also change through “mutations.” A mutation is like if you tried to copy a page from a book but misspelled some words. Mutations usually result from copying errors that occur in certain letters when being passed from parent to child.

When we take a sample of your blood/tissue for this study, it will go to a lab to read off those letters and give us a report on your genome. This information will then be compared with other genomes to see how they may be the same or different. It is our hope this will help us to better understand how the human body works and/or what causes it to not work well, as when someone has a disease.

The tests we might want to use to study your blood and saliva samples may not even exist at this time. Therefore, we are asking for your permission to store your blood and saliva samples so that we can study them in the future. These future studies may provide additional information that will be helpful in understanding Multiple Sclerosis, but it is unlikely that what we learn from these studies will have a direct benefit to you. It is possible that your blood and saliva samples might be used to develop products tests, or discoveries that could be patented and licensed. In some instances, these may have potential commercial value and may be developed by the Investigators, University of Iowa, commercial companies, organizations funding this research, or others that may not be working directly with this research team. However, donors of blood and saliva samples do not retain any property rights to the materials. Therefore, there are no plans to provide financial compensation to you should this occur.

Your blood and saliva samples will be stored with a code which may be linked to your name. If you agree now to future use of your blood and saliva samples but decide in the future that you would like to have it removed from future research, you should contact **Dr. Terry Wahls at 319-356-4421**. However, if some research with your blood and saliva samples has already been completed, the information from that research may still be used.

**WILL I BE NOTIFIED IF MY BLOOD/SALIVA/MRI RESULT(S) IN AN UNEXPECTED FINDING?**

The results from MRI images we collect in this research study are not the same quality as what you would receive as part of your routine health care. The MRI image results will not be reviewed by a physician who normally reads such results. Due to this, you will not be informed of any unexpected findings on these images. Blood results with abnormal Vitamin D findings will be reported to your treating physicians for follow-up. The results of the blood, saliva samples, and MRI images will not be placed in your medical record with your primary care physician or otherwise. If you believe you are having symptoms that may require care, you should contact your primary care physician.

**Genetic Research.** One purpose of this study is to look at genes in your DNA and how they affect health and disease. Genes are the instruction manual for the body. The genes you get from your parents decide what you look like and how your body behaves. They can also tell us a person's risk for certain diseases and how they will respond to treatment.

You are being asked to give **blood and saliva** samples for genetic research. What we learn about you from this sample will not be put in your health record. No one else (like a relative, boss, or insurance company) will be given your test results. Your test results will not be shared with your doctor. Genetic testing will be done on blood we draw and the saliva you collect. **We WILL NOT contact you with any specific results from your analysis.**

To study your DNA and RNA, blood totaling 3 teaspoons will be drawn from a vein in your arm using a needle if you agree to participate in this portion of the study. This blood will be drawn at the same time as the other blood drawn for the study and would add an additional 5 minutes to the blood draw time.

**Genetic Information Nondiscrimination Act (GINA).** A federal law called the Genetic Information Nondiscrimination Act (GINA) generally makes it illegal for health insurance companies, group health plans, and employers of 15 or more persons to discriminate against you based on your genetic information. Based on this new law, health insurance companies and group health plans are prohibited from requesting your genetic information that we get from this research. This means that they may not use your genetic information when making decisions regarding your eligibility for insurance coverage or the amount of your insurance premiums. Be aware that this new federal law will not protect you against genetic discrimination by companies that sell life insurance, disability insurance, or long-term care insurance. The law also does not prohibit discrimination if you are already known to have a genetic disease or disorder.

**Contact for Future Studies.** We would like to contact you to invite you to participate in future studies. To do this, we will keep information on how to reach you (such as mailing address, phone, email) as well as information about your multiple sclerosis diagnosis (such as the type of multiple sclerosis) and general characteristics and demographics including gender and birthdate. If the answer is yes, you would

be contacted about the future studies. Agreeing to participate in this study does not obligate you to participate in any future studies. You would sign a new consent for any future studies.

**Please place your initials in the blank next to Yes or No for the statement below.**
**I would like to be contacted about future studies.**

**__________Yes________No (initials)**

**Online Participant Data Storage.** Your data for this study will be stored electronically in an online REDCap platform. The REDCap platform is managed by the Institute for Clinical and Translational Science at the University of Iowa. Only IRB approved research team members will have access to the REDCap data platform. Each team member will be granted access to the REDCap data system through a secure login. REDCap supports Two-factor authentication using DUO. Two-factor authentication requires something that only the user *knows*, with a second factor, something only the user *has* in their possession, to provide strong identity verification.

In the REDCap data platform, primary data is secured in HCIS Pomerantz Data Center. Data backups are secured in ITS Lindquist Data Center. Operating system security includes secure logins, data encryption at rest, remote system logging and configuration and change management. Data backups are encrypted both in flight and at rest. Copies of data are replicated to the remote data center every 15 minutes. There is 100+ points in time copies of data available at any time. Disaster recovery has been tested. You will have the option to complete some study questionnaires online via REDCap rather than on paper if you prefer to do so.

**Confidentiality of study procedures and study materials.** To protect the integrity of the study it is important that you not share the study materials and procedures with others outside of your household. Please do not share study materials and procedures on social media, internet sites or in any way copy them or send them to others.

## WHAT ARE THE RISKS OF THIS STUDY?

You may experience one or more of the risks indicated below from being in this study. In addition to these, there may be other unknown risks, or risks that we did not anticipate, associated with being in this study.

**There may be a loss of confidentiality.**

**Cognitive Decline.** As with any person with MS, it is possible that you will experience some cognitive decline. If we notice that you have experienced cognitive decline throughout the study, we will ask you to retake the Short Portable Mental Status Questionnaire. If your score has decreased since screening or if your score now falls into the moderate to severe cognitive impairment categories, we will recommend that you visit your treating neurologist. We will also send your neurologist a letter which explains that we have noticed a cognitive decline. This letter will ask your neurologist to determine if you are cognitively suitable to continue participating in the study. You will be able to continue with the study if your neurologist deems it okay. If your neurologist deems you to be unable to continue in the study, the research team will end your participation.

**Surveys.** Filling out surveys is usually low risk. You may be tired or embarrassed if you find the surveys difficult to complete. We will send your surveys prior to the site visits so you have the opportunity to complete the questionnaires at your own pace. Online surveys that we ask you to complete between visits can be filled out at your leisure within 5 days. You may skip any question you do not want to answer. A member of the study team will review the completion of questionnaires for completeness. If there are blank answers, you will be asked if this was left blank on purpose or by mistake.

**Nutrition.** Following the nutritional advice to implement the study diet is expected to be low risk. Following the advice will likely cause weight loss, or more unlikely, weight gain, and possibly undesired weight loss. If you lose too much weight, we will ask you to weigh yourself at home once each week for two weeks (in the morning after urinating and wearing typical street clothes) and report that weight to the study team. If your weight continues to remain low, we will ask you to follow up with your personal physician. We will also ask the study dietitian to contact you by phone to help you increase your calorie intake by suggesting foods that are compliant with the diet you have been assigned.

We may ask you to consume more meat and fats such as nuts and coconut oil that are recommended on the Modified Paleo Elimination (MPE) diet or olive oil on the Time Restricted Olive Oil-based (TROO) Ketogenic diet. You will continue weighing yourself once a week and reporting your weight to the study team. When you weight reaches a safe level, you can discontinue the weekly weights.

You may miss eating foods that are excluded from the study diets. It is possible that you will be asked to eat foods whose taste you do not enjoy at this time. It is also possible, although unlikely, that the food may have an interaction with medicines you are taking. Following the nutritional advice may increase the cost of your weekly grocery bill. Eating meals prepared at home will reduce the expense of implementing the study diet. It is hard to know how the new foods and supplements you will be taking will affect your medications. Those taking Coumadin (warfarin), insulin, or weight loss medication that cause fat malabsorption are not allowed to participate in the study.

It is also possible that your disease will continue to progress, and your various (or new) multiple sclerosis related symptoms will worsen (or develop) while on the study diet.

Because we are informing you of the potential connection between the foods you eat and MS related symptoms, and the potential effects of food on physical symptoms in general, your awareness of new physical symptoms might be heightened by participation in this study. These symptoms may include, but are not limited to, a rash, transient headaches, or muscle or joint pain, which are self-limited and would be expected to resolve within a week.

Other side effects of the diets may include:

- Diarrhea, nausea, bloating and other abdominal complaints
- Change in bowel habits
- Possible development of kidney stones
- change in blood sugar, blood pressure, body weight, blood lipids and dosing requirements for chronic medications
- Foods on the study diet may be more expensive, foods that are recommended may not be familiar and as tasty; foods that are excluded may be familiar comfort foods that are missed.
- It will take more time to prepare home cooked meals.

**Pregnancy**

If you are pregnant, planning to get pregnant, or breastfeeding, you should not enroll in this study. If you can become pregnant and you are sexually active, you are asked to practice an effective method of birth control while in this study. Methods of birth control include total abstinence (no sex), oral contraceptives (pills), an intrauterine device (IUD); Levonorgestrel implants (Norplant), or Medroxy-progesterone acetate injections (Depo-Provera shots). If these cannot be used, contraceptive foam with a condom is recommended. If you are not sure about birth control or whether you could still get pregnant, discuss this with your doctor. Even if you have been told that you are infertile, we ask you to use an effective form of birth control while in the study. That is because adopting either study diet may increase male and female fertility, which could increase the possibility of conceiving. If you do end up getting pregnant over the course of the study, please contact the study team immediately. There is risk of fetal malformation while following the ketogenic diet (in animal models only). You will be advised to follow the dietary and supplement recommendations of the medical team providing your pregnancy care (i.e., obstetrician or family physician).

If you are currently breastfeeding or become pregnant and intend to breastfeed, please contact the study team immediately. You will be advised to follow the dietary and supplement recommendations of the physician providing care to you and/or your baby (i.e. pediatrician or family physician).

There may be an increased risk of ketoacidosis while breastfeeding and following a ketogenic or paleo diet. If you are pregnant or breastfeeding and experience nausea or vomiting, contact your medical team immediately.

If you become pregnant during the study or begin to breastfeed after delivery, we will ask that you continue to complete the surveys and return for your end of study visit at the University of Iowa. We will ask to continue to follow you for the duration of the study even if you discontinue the study diet. Information provided will still be beneficial to the study.

**Coconut oil risk**

- Theoretic increase in total cholesterol
- Rare cases of severe allergic reaction

**Fish Oil**

- May increase the risk of vitamin A and D toxicity if taken at levels much higher than used in the study confusion or unusual excitement; diarrhea; dizziness or drowsiness; double vision; severe headache, irritability, or vomiting; and peeling of skin, especially on the lips or palms. Other risks can include gastrointestinal upset, burping, indigestion, abdominal bloating, or pain.

**Blood Collection**

- Collection of blood samples may cause bruising or bleeding at the site of the blood draw.
- You may feel discomfort from the needle used to draw the blood.
- You may feel dizzy, lightheaded, nausea, or experience fainting.

**Motor Assessments (walk, hand coordination, symbol digit tasks)**

- You may feel tired and or embarrassed about your physical abilities if you find the tasks difficult to complete.

**<u>Visual Acuity and Contrast Sensitivity Testing using the Freiburg Vision Test (FrACT)</u>**

- You may feel fatigued. You will be given the time to rest between tests.

**<u>Email and Text Messaging for Study Communications</u>**

Email and text messaging are common ways to communicate. They may also be convenient ways for the participant and researcher to ask questions and receive answers during the research study. Sending information by email or text message, however, has several risks that you should consider prior to use of email and/or texting. These include, but are not limited to:

- Email/text can be circulated, forwarded, stored electronically and on paper, and sent to unintended recipients. They can be intercepted, altered, forwarded, or used without authorization or detection.
- Email/text senders can easily misaddress an email or text sending information to someone other than the intended recipient.
- Backup copies of email/text may exist even after the sender, or the recipient has deleted his or her copy.
- Employers and on-line services have a right to inspect email transmitted through their systems.
- Emails/texts can be used as evidence in court.
- Email can be used to introduce viruses into computer systems.
- If your email is a family address, other family members may see your messages

**Conditions for use of email and text messaging**

The university and researcher cannot guarantee but will use reasonable means to maintain security and confidentiality of email/text information sent and received. You and the research team must consent to the following conditions:

1. Email/text is not appropriate for urgent or emergency situations. The researcher cannot guarantee that any particular email/text will be read and responded to within a specific time period.
2. Email/text must be concise. You should call the research team or schedule a Zoom meeting if the issue is too complex or sensitive to discuss via email/text.
3. You should not use email/text for communication regarding sensitive medical information.
4. The researcher will not forward participant-identifiable email/texts outside of the University of Iowa and affiliates without your prior written consent, except as authorized
5. The research team and university are not liable for breaches of confidentiality caused by you or any third party.

**Please place your INITIALS in the blank next to Yes or No for the statement below:**
**I have read the instructions above regarding use of email and text communications.**

**_______ Yes      _____No**

**<u>Social Media Applications and Internet</u>**

- There is a higher risk for a potential loss or breach in confidentiality due to the use of social media apps such as Facebook, Instagram, or Twitter.
- Comments posted on social media sites will have the ability to be seen by everyone who has

access to them including but not limited to:
  * Persons provided resources to specific Facebook Pages
  * Persons choosing to or already follow Dr. Wahls' Facebook page
  * Persons choosing to or already follow Dr. Wahls' Instagram
  * Persons choosing to or already follow Dr. Wahls' Twitter account.
- Comments posted on social media sites may cause you to feel uncomfortable, angry, or distressed.
- You may not like how people react to your comments or the other comments posted of others.
- You may not like the answers to questions posted on social media sites.
- You may not like information, articles, or pictures posted social media sites.

**Genetic Research**

One risk of giving samples for this research may be the release of your name that could link you to the stored samples and/or the results of the tests run on your samples. To prevent this, these samples will be given a code. Only the study staff will know the code. The name that belongs to the code will be kept in a locked file or in a computer with a password. Only approved members of the study team will have access to your name.

**MRI Scan**

You may be uncomfortable inside the MRI scanner if you do not like to be in closed spaces ("claustrophobia"). During the procedure, you will be able to talk with the MRI staff through a speaker system. You can tell them to stop the scan at any time.

The MRI scanner produces a loud hammering noise, which has produced hearing loss in a very small number of patients. You will be given earplugs to reduce this risk.

Due to the use of the strong magnet, an MRI cannot be performed on patients with implanted pacemakers, intracranial aneurysm clips, cochlear implants, certain prosthetic devices, implanted drug infusion pumps, neurostimulators, bone-growth stimulators, certain intrauterine contraceptive devices, or other type of iron-based metal implants. Metal objects being attracted to the MRI magnet presents the greatest risk to study participants.

There is also a risk that we will discover an abnormality or disease in the subject, who is otherwise thought to be healthy. This risk can increase the psychologically induced stress associated with participating in a research study. Should an abnormality be discovered, a trained radiologist will look over the images. If they determine further medical examination needs to be done, your doctor will be contacted and in return will get in touch with you. If the radiologist determines nothing needs to be done, you will not be informed as to not induce worry. At the time of the scan, you will not be told if there is an abnormality seen. This is to decrease unnecessary worry.

Although there are no known risks associated with limited exposure to magnetic fields, we cannot rule out the possibility that in the future some risks may be discovered. There is a possibility that the subject will experience vertigo/dizziness or instability when they enter the magnetic field.

Lastly, there is also a risk of a breach of confidentiality.

## WHAT ARE THE BENEFITS OF THIS STUDY?

We don't know if you will benefit from being in this study. However, we hope that, in the future, other people might benefit from this study because it will help determine what eating patterns may be effective for improving fatigue, mood, quality of life, long-term effects on motor function, low-contrast sensitivity, and disease activity assessed by brain imaging in patients that are diagnosed with relapsing remitting multiple sclerosis.

## WHAT OTHER TREATMENT OPTIONS ARE THERE?

Before you decide whether to be in this study, your doctor will discuss the other options that are available to you. The diet in this study is in addition to whatever treatments your doctor has already prescribed. You will continue to take any medications your doctor has prescribed throughout the study.

## WILL IT COST ME ANYTHING TO BE IN THIS STUDY?

You will have costs for being in this research study. Reimbursement for some study expenses will be provided. You will have travel and transportation costs.

You will **NOT** be reimbursed for meals, incidentals, or food costs. You may have a higher food bill as you adopt the study diet. The higher cost may be offset by preparing more home cooked meals.

If you are assigned to 2 of the study diets, you will be dispensed 3 study supplements at your Month 0 / Baseline and Month 3 visit. If you live in the United States, additional study supplements will be mailed to you throughout the duration of the study at no cost to you. You will be asked to bring back empty supplement containers to ensure study participation compliance and correct dispensation.

If you are assigned to 2 of the study diets AND live in Canada or Mexico, at your Month 3 visit you will be asked to take a bag containing study supplements for the duration of the study home with you (~50 bottles). If you are unable to take all bottles back home from your study visit, you will be sent home with about ~20 bottles. In addition, you will be asked to purchase the remaining 30 study supplements, have them shipped to your home, and be reimbursed for the cost. The retail value of these supplements is approximately $1,000. In the event you are unable or unwilling to purchase these supplements for reimbursement, you will not be excluded from the study. There is a chance you will not have study supplements included in your study intervention.

You and/or your medical/hospital insurance carrier will remain responsible for your regular medical care expenses.

## WILL I BE PAID FOR PARTICIPATING?

You will be paid for being in this research study. You may choose to participate without being paid if you choose. You will need to provide your address for a check processed and mailed to you.

The study will reimburse you for your time at each visit held at the University of Iowa and for completing online surveys every 6 months (months 6, 12, 18). You will be paid $100.00 for Month 0 / Baseline, Month 3, and Month 24 / End of Study respectively following the completion of the study assessments and online surveys. You will be paid $25 for completing online surveys every 6 months between the site visits. Payments will be processed every 3-6 months (after site visits occur and bi-annually for online surveys).

The study may reimburse you for your mileage to and from study visits according to the published IRS reimbursement rate (max 500 miles one way based on Google maps) for medical visits. If you park in the hospital ramp, you will need to cover the cost of parking your car during the time of the study visits. If you will travel more than 100 miles to the study visit (200 miles round trip), you can be reimbursed for one night of lodging per study visit according to the published IRS hotel reimbursement rate if you provide us with an itemized hotel/motel bill/folio. Lodging reimbursement is limited to the actual/reasonable cost of a single room plus applicable taxes with a maximum rate of $96.00. Travelers are encouraged to ask for the discounted rate and inquire about free shuttles to the hospital when making reservations (i.e., the government/educational/corporate rate). We will provide you with a list of local hotels and motels.

You will be reimbursed for the purchase of study supplements if you are assigned to 2 of the study diets, live in Canada or Mexico, and are willing to purchase study supplements for Months 13-24.

## DO THE RESEARCHERS HAVE PERSONAL FINANCIAL INTEREST IN THIS STUDY?

The individual leading this research study might benefit financially from this project. Specifically, Dr. Wahls is the owner of a company called Dr. Terry Wahls LLC, which may benefit from the results of this study. In addition, Dr. Wahls has an interest in a branded diet which is subject of this research.

The University's Conflict of Interest in Research Committee has reviewed the financial interest related to this study and developed a plan to manage it. The plan requires that you be informed of the financial interest in this consent form. If you would like more information, please ask the researchers or the study coordinator.

## WHO IS FUNDING THIS STUDY?

The University and the research team are receiving no payments from other agencies, organizations, or companies to conduct this research study.

## WHAT IF I AM INJURED AS A RESULT OF THIS STUDY?

- If you are injured or become ill from taking part in this study, medical treatment is available at the University of Iowa Hospitals and Clinics.
- The University of Iowa does not plan to provide free medical care or payment for treatment of any illness or injury resulting from this study unless it is the direct result of proven negligence by a University employee.
- If you experience a research-related illness or injury, you and/or your medical or hospital insurance carrier will be responsible for the cost of treatment.

## WHAT ABOUT CONFIDENTIALITY?

We will keep your participation in this research study confidential to the extent permitted by law. However, it is possible that other people such as those indicated below may become aware of your participation in this study and may inspect and copy records pertaining to this research. Some of these records could contain information that personally identifies you.

- federal government regulatory agencies,
- auditing departments of the University of Iowa, and
- the University of Iowa Institutional Review Board (a committee that reviews and approves research studies)

To help protect your confidentiality, we will use a study ID and not your name on data collection materials. The file that connects your name and Study ID will be kept in a locked room. Your consents and materials with your name will be kept in a locked room. The electronic data will be kept in the university internal medicine network folder. Network access will be limited to only approved members of the study team. Each study team will access the folder through a secure login. REDCap data entry and storage of participant data for this study will be stored electronically in the REDCap platform. The REDCap platform is managed by the Institute for Clinical and Translational Science at the University of Iowa. Only IRB approved research team members will have access to the REDCap data platform. Each team member will be granted access to the REDCap data system through a secure login.

If we write a report or article about this study or share the study data set with others, we will do so in such a way that you cannot be directly identified.

The University of Iowa Hospitals and Clinics generally requires that we document your participation in research occurring in a University of Iowa Health Care facility. This documentation will be in either your medical record or a database maintained on behalf of the institution reflecting that you are participating in this study. The information included will provide contact information for the research team as well as information about the risks associated with this study. We will keep this Informed Consent Document in our research files; it will not be placed in your medical record chart.

**WILL MY HEALTH INFORMATION BE USED DURING THIS STUDY?**

The Federal Health Insurance Portability and Accountability Act (HIPAA) requires University of Iowa Health Care to obtain your permission for the research team to access or create “protected health information” about you for purposes of this research study. Protected health information is information that personally identifies you and relates to your past, present, or future physical or mental health condition or care. We will access or create health information about you, as described in this document, for purposes of this research study. Once University of Iowa Health Care has disclosed your protected health information to us, it may no longer be protected by the Federal HIPAA privacy regulations, but we will continue to protect your confidentiality as described under “Confidentiality.”

We may share your health information related to this study with other parties including federal government regulatory agencies, the University of Iowa Institutional Review Boards and support staff.

You cannot participate in this study unless you permit us to use your protected health information. If you choose *not* to allow us to use your protected health information, we will discuss any non-research alternatives available to you. Your decision will not affect your right to medical care that is not research related. Your signature on this Consent Document authorizes University of Iowa Health Care to give us permission to use or create health information about you.

Although you may not be allowed to see study information until after this study is over, you may be given access to your health care records by contacting your health care provider. Your permission for us to access or create protected health information about you for purposes of this study has no expiration date. You may withdraw your permission for us to use your health information for this research study by sending a written notice to **Terry Wahls MD** at **200 Hawkins Drive SE636 GH, Iowa City, Iowa 52242.** However, we may still use your health information that was collected before withdrawing your permission. Also, if we have sent your health information to a third party, such as the study sponsor, or

we have removed your identifying information, it may not be possible to prevent its future use. You will receive a copy of this signed document via email. You can download a copy of this signed document via REDCap also.

## IS BEING IN THIS STUDY VOLUNTARY?

Taking part in this research study is completely voluntary. You may choose not to take part at all. If you decide to be in this study, you may stop participating at any time. If you decide not to be in this study, or if you stop participating at any time, you won't be penalized or lose any benefits for which you otherwise qualify.

### What if I Decide to Drop Out of the Study?

If you decide to leave the study early, we will ask you to tell us why you have chosen to leave the study before completing. The study team will ask what tasks you will be willing to complete including online surveys, so we have as much data as possible about your participation. If you are willing to come for an in-person visit, we will also complete the physical assessments as well. If you are not able to return in person, we would still appreciate online questionnaires to still be completed.

### Will I Receive New Information About the Study while Participating?

If we obtain any new information during this study that might affect your willingness to continue participating in the study, we'll promptly provide you with that information.

### Can Someone Else End my Participation in this Study?

Under certain circumstances, the study team may decide to end your participation in this research study earlier than planned. This could happen because in the judgment of the study team it would not be safe for you to continue in the study or because funding for the study has ended. If you are not able or willing to provide information about the foods that you are adding and limiting in your diet the study team may decide to end your participation at that time. Because there are only two visits held at the site the online survey data and participation is an important part of the study. If the study team observes lack of participation, they may determine that your participation in the study should end. Lastly, if inappropriate comments are posted on study specific online platforms or during group calls, the study team may decide to end your participation from the study.

## WHAT IF I HAVE QUESTIONS?

We encourage you to ask questions. If you have any questions about the research study itself, please contact: the clinical coordinator, Mary Ehlinger at 319-384-5002. If you experience a research-related injury, please contact: Dr. Terry Wahls at 319-356-4421. If you have questions, concerns, or complaints about your rights as a research subject or about research related injury, please contact the Human Subjects Office, 105 Hardin Library for the Health Sciences, 600 Newton Rd, The University of Iowa, Iowa City, IA 52242-1098, (319) 335-6564, or e-mail irb@uiowa.edu. General information about being a research subject can be found by clicking "Info for Public" on the Human Subjects Office web site, http://hso.research.uiowa.edu/. To offer input about your experiences as a research subject or to speak to someone other than the research staff, call the Human Subjects Office at the number above.

---

This Informed Consent Document is not a contract. It is a written explanation of what will happen during the study if you decide to participate. You are not waiving any legal rights by signing this

Informed Consent Document. Your signature indicates that this research study has been explained to you, that your questions have been answered, and that you agree to take part in this study. You may download a copy of this form.

Subject's Name (printed): ____________________________________________

______________________________ ______________________________
(Signature of Subject) (Date)

**Statement of Person Who Obtained Consent**

I have discussed the above points with the subject or, where appropriate, with the subject's legally authorized representative. It is my opinion that the subject understands the risks, benefits, and procedures involved with participation in this research study.

______________________________ ______________________________
(Signature of Person who Obtained Consent) (Date)
