# Supplementary material for: Efficacy of Diet on Quality of life in Multiple Sclerosis (EDQ-MS): a study protocol for a randomized controlled clinical trial
Source: Trials. 2025 Oct 27;26:437. doi: 10.1186/s13063-025-09157-2 (PMC12557952; doi:10.1186/s13063-025-09157-2)
Supplement: Supplementary file 2 — Supplementary Material 2 [file 13063_2025_9157_MOESM2_ESM.pdf]

1. **Canvas Classroom (Intervention groups only)**: Study participants in the intervention arms will be given access to University of Iowa's Canvas Classroom to be used as a focus group and discussion platform. This account will be offered only to study participants; however, all participants assigned to the MPE and TROO Ketogenic diet intervention arms will be given access to different classrooms. The participants will be invited to share their experiences, post recipes, and questions on the classroom discussion page. Participant resources will be added to the classroom so participants will have the option to access if desired. Participants will be informed that Canvas Classroom is completely voluntary and not required as part of participating in the study. The participants can see the online user list in Canvas and may reach out to one another on this platform. If a participant is contacted and doesn't want to engage, they can choose not to respond to a request. At the end of the study, Canvas analytics can be used to track participant engagement and performance metrics.
2. **MyCap Application (Intervention groups only)**: Participants assigned to the intervention will be asked to answer three questions about diet specific components and perceived adherence:

MPE Diet	*TROO Ketogenic Diet*
1. Did you eat gluten containing foods? (yes / no)	1. What was your ketone level? (not tested, < 0.5; 0.5-1.0; 1.1-3.9, ≥4)
2. How many cups (measured raw) of non-starchy vegetables did you eat? (0, 1, 2, 3, 4, 5, 6, 7, 8, 9+)	2. Did you consume at least 2 Tbsp. of olive oil? (yes / no)
3. Rating of diet adherence? (Excellent, good, fair, poor)	3. Rating of diet adherence? (Excellent, good, fair, poor)

3. **EDQMS Application: (Intervention groups only):** Participants will be offered an additional resource developed for this study to include a recipe and meal planning application (designed by 3rd party, FOOGAL) if the study participant is assigned the MPE diet or TROO Ketogenic

diet. The study participant will only be given access to recipes that are consistent with the key components of the study diet they are assigned. This application is free to download, is NOT required for participation in the study, and does not collect any subject data. The purpose of the EDQMS application is to provide assistance, resources, and support for meal planning and shopping during the study only. A non-identifiable email address will be created by the research team to protect the identity of the study participant. An invitation will be sent to this address for approval, set-up, and access to the application. There will be no direct interaction or data collection between the study participant and 3rd party developing the application.

4. **USDA Start Simple with MyPlate application (usual care control group)**

Participants assigned to the usual care control group will maintain their current dietary habits and receive educational materials based on the 2020-2025 USDA Dietary Guidelines for Americans (DGA) for a period of two years. To enhance accessibility to these resources, participants will be guided to install the USDA Start Simple with MyPlate mobile application on their phones. This app provides practical tools and resources aligned with the DGA, offering personalized nutrition guidance and tips for maintaining a healthy diet. Additionally, study participants randomized to the usual care group will receive monthly emails and/or text messages to receive resources with articles, websites, recipes, DGA education materials, and recent MS-related research that does not involve diet.
