# Supplementary material for: Efficacy of Diet on Quality of life in Multiple Sclerosis (EDQ-MS): a study protocol for a randomized controlled clinical trial
Source: Trials. 2025 Oct 27;26:437. doi: 10.1186/s13063-025-09157-2 (PMC12557952; doi:10.1186/s13063-025-09157-2)
Supplement: Supplementary file 4 — Supplementary Material 4 [file 13063_2025_9157_MOESM4_ESM.pdf]

**IOWA**
**HEALTH CARE**

***Department of Internal Medicine***
*Division of General Internal Medicine*
*200 Hawkins Drive, SE600 GH*
*Iowa City, IA 52242*
*319-356-4241* ***Tel***
*319-356-3086* ***Fax***
*medicine.uiowa.edu*

August 19, 2024

Editorial Office, *Trials*

Re: TRLS-D-24-01051, Shemirani et al.

To the Editorial Office:

Thank you for your email of August 14 regarding the above-noted submission. This letter is to confirm that the clinical trial titled "Efficacy of Diet on Quality of Life in Multiple Sclerosis (EDQ-MS)" was financially supported through the discretionary funds allocated to me as the Principal Investigator, Dr. Terry L. Wahls. I am using funds from the Wahls Therapeutic Lifestyle Fund which provides unrestricted support to research conducted by Dr. Terry Wahls (me) and her (my) team at the Department of Internal Medicine within the University of Iowa Roy J. and Lucille A. Carver College of Medicine.

Approval to conduct the study "Efficacy of Diet on Quality of Life in Multiple Sclerosis (EDQ-MS)" was given by the University of Iowa Institutional Review Board on December 9, 2021 and I approved use of the funds from the Wahls Therapeutic Lifestyle Fund for paying costs associated with conducting this study. Ms. Lori Bassler is the administrative officer in the Department of Internal Medicine who assists principal investigators with managing research-related budgets has confirmed funds have been allocated and will support this study.

Sincerely,

Terry L Wahls

Terry Wahls MD, IFMCP, FACP
Clinical Professor of Medicine
Departments of Internal Medicine and Neurology
University of Iowa
terry-wahls@uiowa.edu

Loretta Bassler

Loretta Bassler
Director, Research Administration & Financial Operations
Department of Internal Medicine
University of Iowa
lori-bassler@uiowa.edu
